# Update Speicheldrüsenchirurgie

**DOI:** 10.1007/s00106-026-01723-7

**Published:** 2026-02-12

**Authors:** Sarina K. Müller, M. Koch

**Affiliations:** Hals-Nasen-Ohren-Klinik, Kopf- und Halschirurgie, Universitätsklinikum Erlangen, Waldstraße 1, 91054 Erlangen, Deutschland

**Keywords:** Entzündung, Minimal invasive Operationsverfahren, Chirurgische Operationsverfahren, Tumoren, Neck-Dissection, Inflammation, Minimally invasive surgical procedures, Surgical procedures, operative, Neoplasms, Neck dissection

## Abstract

**Hintergrund und Ziel:**

In den letzten Jahren sind auf dem Gebiet der Speicheldrüsenchirurgie Studien publiziert worden, die einen großen Einfluss auf die tägliche Praxis haben. Dies gilt einerseits für die chirurgische Therapie von benignen und malignen Tumoren, andererseits auch für die Behandlung von obstruktiven und inflammatorischen Erkrankungen der Speicheldrüsen. Bei den Tumoren gab es mit der extrakapsulären Dissektion in den letzten 20 Jahren einen Shift zu einer Deeskalation der Fazialisdarstellung und einer Resektion mit einer geringen Morbidität. Bei den entzündlichen und obstruktiven Erkrankungen haben sich minimal invasive Therapiemethoden seit den letzten 35 Jahren, insbesondere aber seit den letzten 10 Jahren durchgesetzt. Ziel der Arbeit ist es, ein Update über die Speicheldrüsenchirurgie zu geben. Dabei beschäftigt sich Teil 1 mit der Chirurgie von Tumoren und Teil 2 mit der Chirurgie von obstruktiven und inflammatorischen Erkrankungen.

**Materialien und Methoden:**

Es handelt sich um eine Zusammenstellung der aktuellen Entwicklungen anhand der neuesten Literatur mit Fokus auf die letzten 10 Jahre und von eigenen Erfahrungen.

**Ergebnisse:**

Die extrakapsuläre Dissektion ist der superfiziellen Parotidektomie bei geeigneter Indikation in den Punkten Frey-Syndrom, Op.-Dauer, ästhetischer Aspekt sowie transiente und permanente Fazialisfunktion überlegen. Bei malignen Tumoren ohne Infiltration des N. facialis sollte der N. facialis im Sinne einer kompletten Parotidektomie erhalten werden. Eine radikale Parotidektomie ist nur bei Fazialisinfiltration indiziert. Eine elektive ipsilaterale Neck-Dissection ist bei T3/4-Tumoren, High-Grade-Karzinomen und adenoidzystischen Karzinomen indiziert. Die therapeutische und interventionelle Sialendoskopie und sialendoskopisch assistierte operative Verfahren dominieren die Therapie bei obstruktiven und inflammatorischen Speicheldrüsenerkrankungen und können auch bei Verletzungen des Speichelgangs von Bedeutung sein.

**Schlussfolgerung:**

Die Indikationsstellung zur Operation und die Wahl der chirurgischen Technik sind von der Lage und der Dignität des Tumors abhängig. Aufgrund der skizzierten Entwicklungen ist eine Resektion der großen Kopfspeicheldrüsen bei obstruktiven bzw. inflammatorischen Erkrankungen in weniger als 5 % der Fälle indiziert.

## Abstract (en)

**Background and objective:**

Recent years have seen publication of studies on salivary gland surgery with a major impact on daily practice. This applies to both the surgical treatment of benign and malignant tumors and to the treatment of obstructive and inflammatory diseases of the salivary glands. In the case of tumors, extracapsular dissection has led to a shift toward de-escalation of facial exposure and resection with low morbidity over the past 20 years. For inflammatory and obstructive diseases, minimally invasive treatment methods have become established over the past 35 years, especially over the past 10. This paper provides an update on salivary gland surgery. Part 1 deals with tumor surgery and part 2 with the surgical therapy of obstructive and inflammatory diseases.

**Materials and methods:**

This article represents a compilation of current developments based on the latest literature, with a focus on the past decade and the authors’ our own experience.

**Results:**

Extracapsular dissection is superior to superficial parotidectomy in terms of Frey syndrome, operating time, esthetic appearance, and transient and permanent facial function—provided the indications are appropriate. In malignant tumors without infiltration of the facial nerve, the latter should be preserved in the context of complete parotidectomy. Radical parotidectomy is only indicated in cases of facial nerve infiltration. Elective ipsilateral neck dissection is indicated for T3/4 tumors, high-grade carcinomas, and adenoid cystic carcinomas. Therapeutic and interventional sialendoscopy and sialendoscopy-assisted surgical procedures dominate the treatment of obstructive and inflammatory salivary gland diseases and can also be important in cases of salivary duct injury.

**Conclusion:**

The indication for surgery and the choice of surgical technique depend on the location and biological behavior of the tumor. Based on the developments outlined above, resection of the major salivary glands is indicated in less than 5% of cases of obstructive or inflammatory diseases.

## Teil 1: Update der Chirurgie von Speicheldrüsentumoren

### Gutartige Parotistumoren

#### Operationsplanung

Bis 2017 hat die Weltgesundheitsorganisation (WHO) 11 und einschließlich des aktuellen Updates 15 benigne epitheliale Speicheldrüsentumoren anerkannt [1, 2]. Die beiden häufigsten gutartigen Tumoren in den großen Kopfspeicheldrüsen sind die pleomorphen Adenome und die Warthin-Tumoren. Weitere gutartige Histologien umfassen u. a. Onkozytome, Basalzelladenome und monomorphe Adenome. Während seit den 1980er-Jahren das pleomorphe Adenom als häufigste gutartige Entität angegeben wurde [3], wird bei monozentrischen Analysen in den letzten Jahren häufiger ein Warthin-Tumor gefunden [4]. Letztendlich ist für die Unterscheidung dieser Tumoren der pathohistologische Befund entscheidend. Für die Operationsplanung allerdings ist es wichtig, schon präoperativ einen Anhalt zu haben, um welche Dignität es sich handelt und welche chirurgische Vorgehensweise voraussichtlich verwendet wird. Dies ist sowohl für die Beratung von Patientinnen und Patienten sehr wichtig als auch für die Organisation des Operationsplans.

Erste Modalität zur Diagnostik von Tumoren der großen Kopfspeicheldrüsen ist und bleibt die B‑Bild-Sonographie [5]. Sonographische Merkmale wie Echogenität, Begrenzung, Perfusion und Invasion können Hinweis auf die Dignität geben. Das Verhältnis des Tumors im Vergleich zur V. retromandibularis kann Aufschluss auf die Tiefe des Tumors geben. Tumoren, die einen Abstand zur Faszie von über 3 mm haben, liegen ebenfalls häufig im tiefen Lappen [6]. Die Kombination aus Demografie und sonographischer Lage kann in einigen Fällen sogar Hinweise auf die genaue Entität der gutartigen Tumoren hinweisen (z. B. bei älteren Männern mit Nikotinabusus mit einem kaudal gelegenen Knoten als Hinweis auf einen Warthin-Tumor). Ein kontrastmittelverstärkter Ultraschall (CEUS) bzw. eine Elastographie können die Vorhersagekraft noch weiter erhöhen. In Fällen, bei denen die Sonographie atypische Befunde zeigt oder bei tiefen bzw. multiplen Tumoren ist eine Magnetresonanztomographie (MRT) indiziert [7].

Die Frage nach der Indikation der präoperativen Probe (Grobnadelbiopsie, Feinnadelbiopsie) wird kontrovers diskutiert und soll in ihrer Breite nicht dargestellt werden. Hauptargument gegen eine präoperative Probenentnahme mittels Grobnadelbiopsie ist allerdings, dass bei einem pleomorphen Adenom die Kapsel durch die Probe eröffnet wird. Durch Streuung von Zellen entlang des Stichkanals können selten Rezidive auftreten. Die Wahrscheinlichkeit liegt hierbei höher als bei Entfernung des Tumors mit einem Randsaum (s. Abschnitt zur chirurgischen Therapie bei Rezidiven von pleomorphen Adenomen). Zum präoperativen Standard gehört ebenfalls die Klassifikation der Fazialisfunktion mittels Scores (z. B. House-Brackmann) und die Durchführung einer Elektromyographie.

#### Benigne Tumoren in der Gl. parotidea

**Abb. 1:**
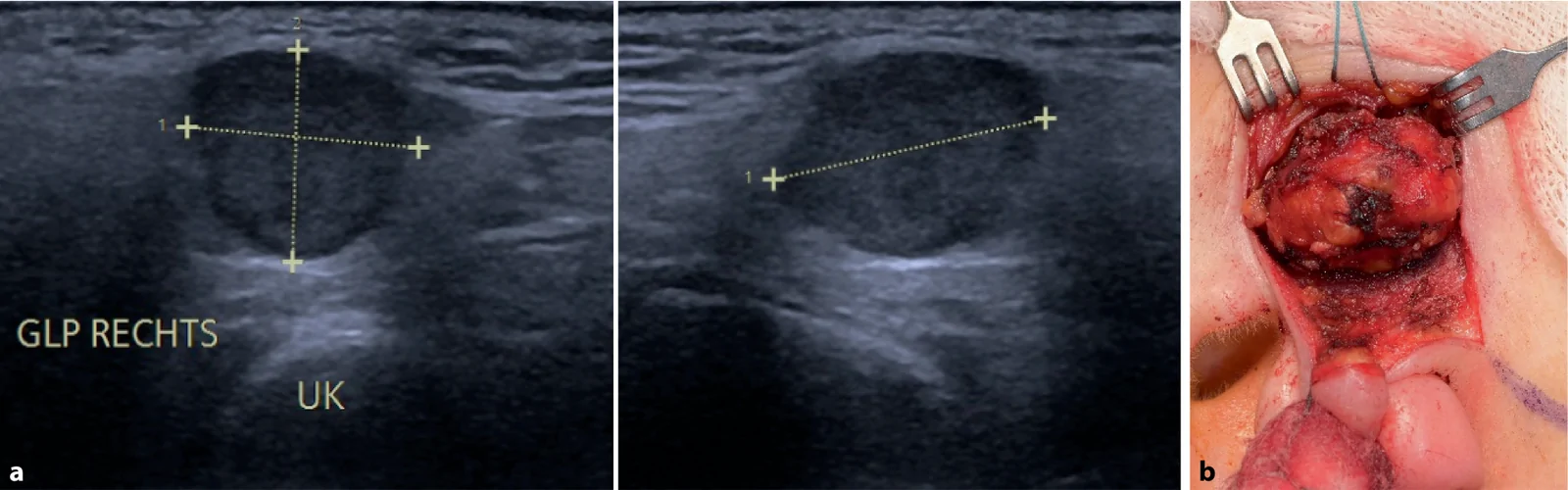
**a** B-Bild-Sonographie eines Tumors der rechten Gl. parotidea (*GLP*). **b** Dazugehöriges intraoperatives Foto einer extrakapsulären Dissektion des histologisch gesicherten pleomorphen Adenoms. *UK* Unterkiefer

Historisch ist die Chirurgie der Goldstandard der Therapie von Tumoren der großen Kopfspeicheldrüsen. Dies hat sich bis zum heutigen Tag auch nicht geändert. Was sich aber über die letzten Jahre und Jahrzehnte geändert hat, sind die chirurgischen Techniken. Waren einst die superfizielle Parotidektomie der alleinige internationale Goldstandard für die Entfernung gutartiger Tumoren im oberflächlichen Lappen und eine subtotale bis totale Parotidektomie für gutartige Tumoren im tiefen Lappen, so ist die extrakapsuläre Dissektion (ED; Abb. 1) des Tumors national und international ebenfalls eine primäre Therapieform bei definierten Tumoren und nach korrekter Indikationsstellung [8–11].

**Abb. 2:**
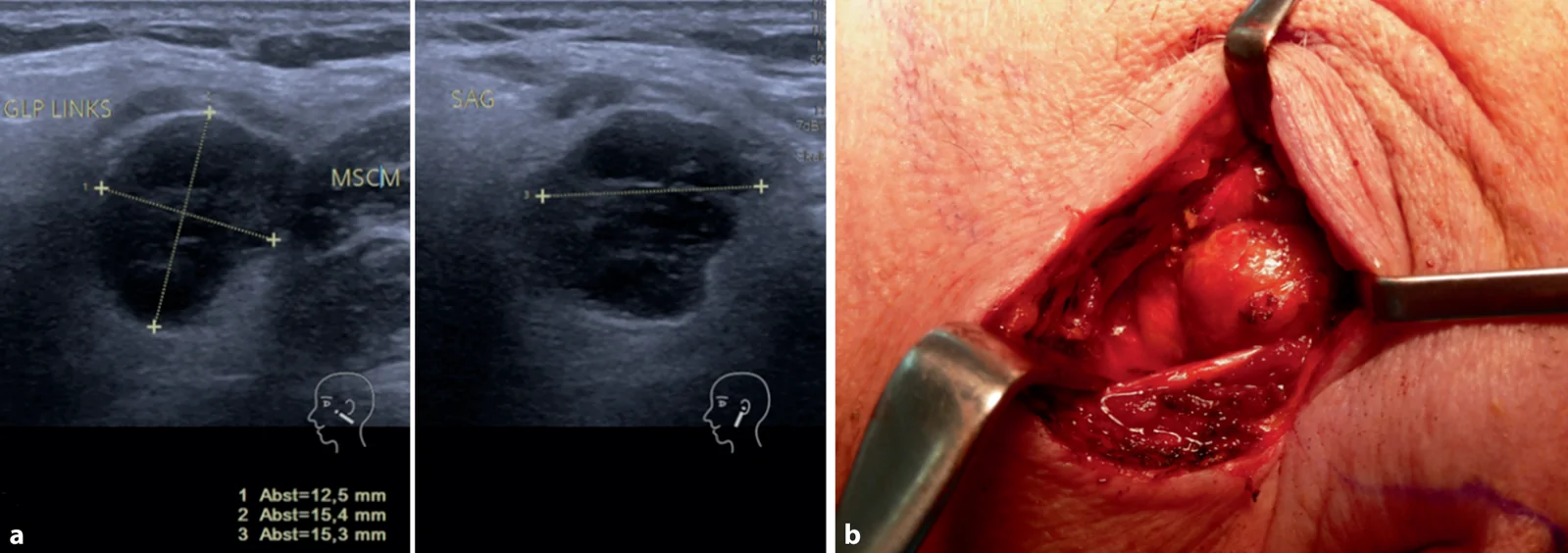
**a** B-Bild-Sonographie eines Tumors der linken Gl. parotidea (*GLP*). **b** Dazugehöriges intraoperatives Foto einer (extra)kapsulären Dissektion des histologisch gesicherten Warthin-Tumors. *MSCM *M. sternocleidomastoideus,* SAG* Sagittal

Die ED ist definiert als Entfernung des Tumors mit einem belassenen Gewebesaum um die Tumorkapsel [12]. Definitionsgemäß wird der Hauptstamm nicht dargestellt, so wie es bei der superfiziellen oder kompletten Parotidektomie der Fall ist. Bei der ED werden sowohl die Drüsenkapsel als auch das -parenchym eröffnet und nur der Tumor mittels eines Gewebesaums entfernt unter Belassung der Restdrüse. Ein Sicherheitssaum von 4–5 mm wird empfohlen. Diese Empfehlung basiert auf verschiedenen Veröffentlichungen, die zeigten, dass Satellitentumoren von pleomorphen Adenomen bis zu einem Abstand von durchschnittlich 3–4 mm (2,3 bis maximal 8,5 mm) von der Tumorkapsel im histologischen Präparat nachgewiesen werden können [13]. Im Fall der rein kapsulären Dissektion des kompletten Tumors würden diese Anteile als Residuum belassen werden. Dies trifft allerdings ausschließlich auf pleomorphe Adenome zu. Bei Warthin-Tumoren kann auch eine kapsuläre Dissektion erfolgen, d. h. eine Dissektion um die Tumorkapsel herum, bei der nicht 4–5 mm Parotisgewebe um den Tumor belassen wird ([14]; Abb. 2). Dies begründet sich aus histopathologischen Kriterien wie dem Nichtvorhandensein von Satellitentumoren. Obwohl für unklare Tumoren und Tumoren, bei denen ein pleomorphes Adenom vermutet wird, 4–5 mm Sicherheitsabstand empfohlen werden, ist dieser Abstand praktisch nicht immer möglich. Wenn der Tumor relativ oberflächlich liegt, d. h. in Drüsenkapselnähe bzw. an weiteren anatomischen Strukturen wie dem M. sternocleidomastoideus, dann ist das Belassen eines größeren Saums von Parotisgewebe nicht möglich. Das gleiche gilt auch für Tumoren, an oder in deren Kapsel der N. facialis läuft. In diesem Fall muss an den Stellen, an denen der Nerv in unmittelbarer Nähe zu oder sogar in der Tumorkapsel läuft, zur Nervenschonung eine kapsuläre Dissektion erfolgen. An den Stellen der kapsulären Dissektion entsteht eine „bare area“. Hierbei ist zu beachten, dass diese „bare area“, unabhängig von der Op.-Technik, allein aufgrund der Nähe des Nervs zum Tumor besteht [15]. Dies bedeutet, dass eine kapsuläre Dissektion auch bei einer superfiziellen oder kompletten Parotidektomie erfolgen muss [16].

Wichtig bei der ED ist v. a. die richtige Indikationsstellung. Die Indikation für eine ED besteht insbesondere bei singulären Tumoren, die gut mobil sind und im oberflächlichen Lappen liegen. In der Bildgebung weisen sie benigne Kriterien auf. In erfahrenen Händen können aber auch tiefer gelegene und nicht sehr mobile Tumoren mittels einer ED entfernt werden [17]. Die technische Möglichkeit und Erfahrung des Operateurs oder der Operateurin zum Umschwenken auf eine ausgedehntere Resektion mit Hauptstammdarstellung sollte jedoch jederzeit gegeben sein. Während auch hier eine ED zumindest technisch möglich ist, ist besondere Vorsicht geboten, um den Stenon-Gang nicht zu verletzen. In diesem Fall kann eine Gangschienung (z. B. mittels einer Myrthenblattsonde oder sialendoskopisch kontrollierter Einführung eines Stents) erfolgen, um den Gang intraoperativ besser identifizieren zu können. Im Zweifelsfall sollte die operierende Person in der Lage sein, intraoperativ auf ausgedehntere Resektionsformen umzusteigen, wenn dies sich als die sicherere Variante erweist. Prinzipiell gilt, dass eine ED umso einfacher und besser durchzuführen ist, je weiter inferior und posterior der Tumor gelegen ist.

Bei einzelnen, nicht mobilen und tiefen Tumoren sollte immer auch erwogen werden, die superfizielle Parotidektomie durchzuführen. Hierbei wird dann der Hauptstamm des N. facialis dargestellt und sowohl das zerviko- als auch das temporofaziale Bündel nach distal verfolgt, sodass der laterale Drüsenlappen entfernt werden kann. Bei Tumoren in oberflächlichen Lappen und nach Hauptstammdarstellung kann für ein besseres ästhetisches Ergebnis auch eine partiell superfizielle Operation durchgeführt werden. In diesem Fall wird der Hauptstamm dargestellt, aber nur der Teil des oberflächlichen Lappens entfernt, in dem der Tumor liegt. Das restliche oberflächliche Parotisgewebe wird belassen.

**Abb. 3:**
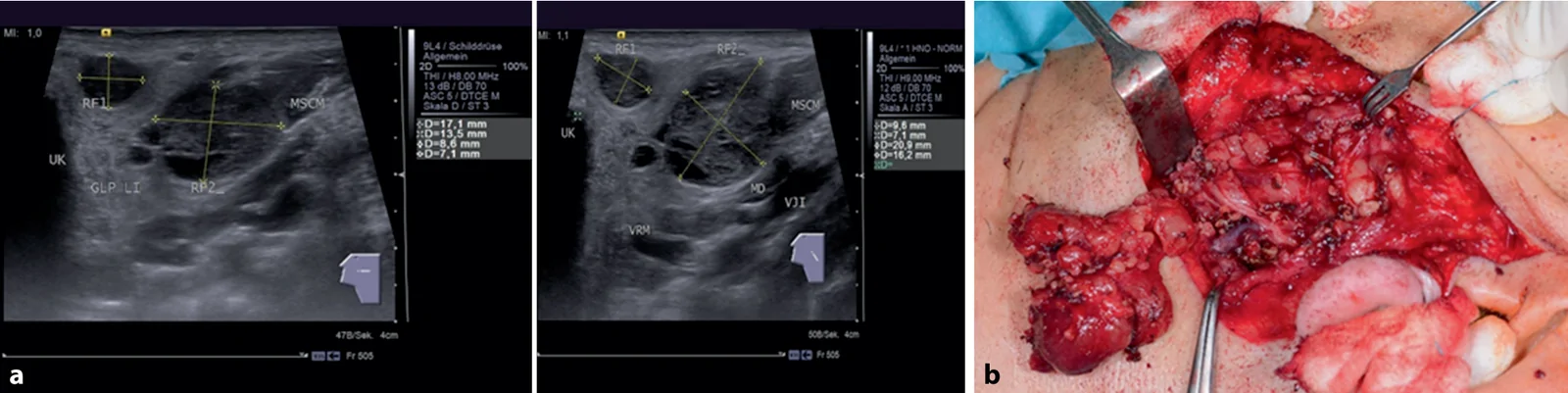
**a** B-Bild-Sonographie eines multilokulären Warthin-Tumors der linken Gl. parotidea (*GLP LI*). **b** Dazugehöriges intraoperatives Foto einer partiellen Parotidektomie aller Nodi des histologisch gesicherten Warthin-Tumors. *Gl. *Glandula, *li * links, *MSCM *M. sternocleidomastoideus, *RF* Raumforderung, *UK* Unterkiefer

**Abb. 4:**
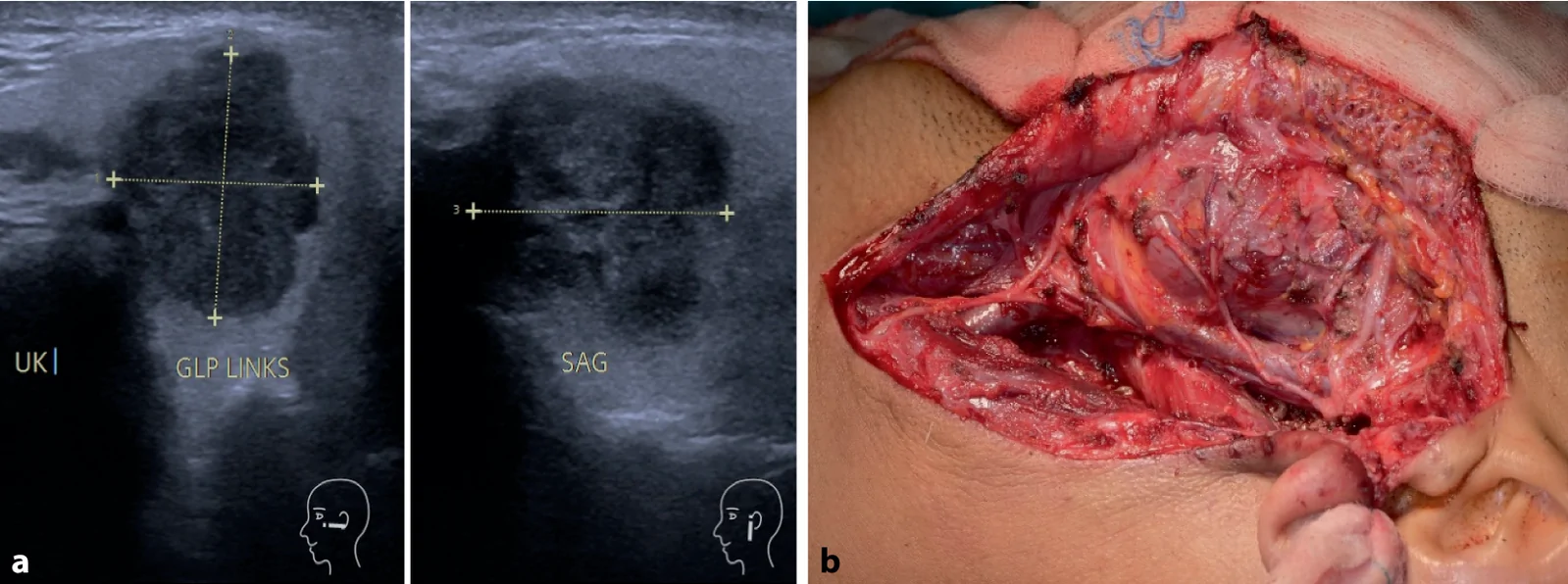
**a** B-Bild-Sonographie eines Tumors der linken Gl. parotidea (*GLP*). **b** Dazugehöriges intraoperatives Foto einer kompletten Parotidektomie des histologisch gesicherten Mukoepidermoidkarzinoms. *SAG* Sagittal, *UK* Unterkiefer

Bezüglich des Outcomes gibt es mehrere Übersichtsarbeiten bzw. Metaanalysen, die die superfizielle Parotidektomie mit der extrakapsulären Dissektion vergleichen [11, 17–22]. Randomisierte kontrollierte Studien sind nicht vorhanden. In diesen werden unter anderen die Op.-Dauer, die Häufigkeit des postoperativen Frey-Syndroms, der ästhetische Aspekt sowie die transiente und permanente Fazialisfunktion verglichen. In allen Punkten wurde die extrakapsuläre Dissektion als bevorzugt gewertet. In allen Studien erwies sich die ED als vorteilhafter. Beispielsweise wurden in einer Metaanalyse 14 Kohortenstudien mit insgesamt 3194 Patientinnen und Patienten verglichen [19]. Das gepoolte relative Risiko (RR) hat dabei keinen signifikanten Unterschied zwischen den Rezidivraten im Vergleich von superfiziellen Parotidektomie und ED ergeben (Fixed-Effect-Modell: RR = 0,71; 95 %-Konfidenzintervall, 95 %-KI: 0,40–1,27; *p* = 0,249; Random-Effect-Modell: RR = 0,67; 95 %-KI: 0,38–1,23; *p* = 0,197). Das Follow-up der verschiedenen Studien lag zwischen 6 Monaten und 30 Jahren. Die Berechnung eines gemittelten Follow-up ist nicht möglich, da die verschiedenen Studien entweder den Mittelwert oder den Median angeben. Es sind 2 Studien eingeschlossen worden, die ein sehr kurzes Follow-up (Spannweite: 0–2 Jahre, Mittelwert 18 Monate) hatten. 6 Studien hatten ein Follow-up von über 10 Jahren (Spannweite: 3–30 Jahre). Bei 5 Studien lag das Follow-up zwischen 2 und 10 Jahren. Bei einer eingeschlossenen Studie war das Follow-up unklar. Ein längeres Follow-up, aufgeschlüsselt nach Op.-Technik, ist nötig, um auch im Langzeitverlauf die Rezidivraten vergleichen zu können. Bei der ED wurden in 15,7 % der Fälle Sialozelen beschrieben, die in 87,5 % innerhalb eines Monats nicht mehr darzustellen waren. In anderen Studien sind diese vergleichsweise niedrig mit einer Häufigkeit von 2,4–13,3 % [20, 22]. Eine komplette Parotidektomie bei gutartigen Tumoren sollte im Fall einer Gangverletzung durchgeführt werden, um Sialozelen zu vermeiden. Auch bei multiplen Tumoren, z. B. im Rahmen eines multilokulären Warthin-Tumors, sollte eine komplette Drüsenentfernung erwogen werden. Bei mehreren Knoten, die inferior posterior liegen und sich gut verschieben lassen, können alle Tumoren allerdings auch mittels ED oder partieller Parotidektomie en bloc entfernt werden (Abb. 3; [10, 11]). Bei der kompletten Parotidektomie werden beide Lappen der Gl. parotidea komplett entfernt. Das bedeutet auch, dass der N. facialis, der den oberflächlichen und tiefen Lappen trennt, komplett dargestellt und skelettiert wird (Abb. 4). Der Stenon-Gang wird unterbunden oder geklippt. Die Notwendigkeit einer kompletten Parotidektomie ist auch bei Innenlappentumoren mit erfolgter kompletter Nervendarstellung gegeben. Bei Innenlappentumoren, die so inferior unter dem zervikofazialen Bündel liegen, dass nicht der komplette Fazialisfächer dargestellt werden muss, ist eine extrakapsuläre Dissektion möglich. Hierbei ist die Darstellung des Vorderrands des M. sternocleidomastoideus und des Venter posterior des M. digastricus der Schlüssel, um den Innenlappentumor von kaudal extrakapsulär entfernen zu können. Eine weitere seltene Ausnahme, in der bei Innenlappentumoren keine komplette Parotidektomie notwendig ist, stellen im Schnellschnitt nachweislich gutartigen Tumoren und hier v. a. pleomorphe Adenome dar. Pleomorphe Adenome sind häufig in der oder unterhalb der Fazialisbifurkation und somit im Innenlappen lokalisiert. Zur Entfernung dieser Tumoren ist eine Darstellung des Fazialisfächers soweit notwendig, dass der Tumor entfernt werden kann. Bei gutartiger Histologie kann der oberflächliche Anteil aus ästhetischen und funktionellen Gründen wieder zurückgeklappt und somit auf eine komplette Parotidektomie verzichtet werden. Die komplette Parotidektomie weist die höchste Rate an temporären und permanenten Fazialisparesen auf (Odds Ratio, OR: 4,27 gegenüber der lateralen Parotidektomie für temporäre Fazialisparese und OR 4,29 gegenüber der lateralen Parotidektomie für permanente Fazialisparesen [21]).

Für alle beschriebenen Techniken ist ein Nervenmonitoring des N. facialis Standard. Hierfür werden jeweils 2 Elektroden in den M. orbicularis oculi und den M. orbicularis oris gestochen. Während bei der superfiziellen und kompletten Parotidektomie auch anatomische Landmarken genutzt werden können, verlässt sich die extrakapsuläre Dissektion auf das Nervenmonitoring. Begonnen werden sollte mit einer maximalen Stromstärke von 4 mA. Bei Identifikation und Darstellung des Nervs sollte diese auf 1 mA reduziert werden [22].

In einer Metaanalyse von 546 Patientinnen und Patienten aus 7 Studien wurde gezeigt, dass die direkte postoperative Fazialisfunktion signifikant besser war in der Gruppe, die mit Nervenmonitoring operiert worden war, im Vergleich zu der Gruppe, die nicht mit Nervenmonitoring operiert worden war (22,5 vs. 34,9 %; *p* = 0,001). Die Rate an permanenten Fazialisparesen erwies sich auch in der Gruppe, die mit Nervenmonitoring operiert worden war, als geringer, jedoch ohne statistische Signifikanz (3,9 vs. 7,1 %; *p* = 0,18) [23]. Auch eine weitere Metaanalyse zeigt ähnliche Ergebnisse. Bei der Analyse von 10 Studien mit 1069 Patientinnen und Patienten erwies sich die Rate an direkten postoperativen (23,4 vs. 38,4 %; *p* = 0,001) sowie an permanenten Fazialisparesen (5,7 vs. 13,6 %; *p* = 0,001) als signifikant niedriger bei der Gruppe, die mittels Fazialismonitoring operiert worden war. In einer Subgruppenanalyse der prospektiv durchgeführten Studien gab es jedoch für beide Szenarien keinen signifikanten Unterschied [24]. Standardmäßige Schnittführung ist die modifizierte Blair-Inzision, die jeweils nach Lage des Tumors und Op.-Technik in Länge und Lage angepasst werden sollte, um ein ästhetisch möglichst ansprechendes Ergebnis zu erreichen.

#### Benigne Tumoren der Gl. submandibularis und der Gl. sublingualis

Während bei der Operation an der Gl. parotidea die Entfernung der kompletten Drüse bei gutartigen Befunden nur selten nötig ist, stellt die komplette Drüsenentfernung weiterhin den Goldstandard bei Tumoren der Gl. submandibularis und sublingualis dar. Allerdings wurden auch in der Gl. submandibularis erste Fallserien publiziert, die eine extrakapsuläre Dissektion beschreiben [25]. Bei einem maximalen Follow-up von 6 Jahren ist aktuell aber noch nicht abzuschätzen, ob keine vermehrten Rezidive bei dieser Technik auftreten oder ob das Follow-up noch zu kurz ist. In Europa bleibt die submandibuläre Inzision Schnittführung der Wahl. Andere Techniken wie ein endoskopisches Vorgehen durch eine Haarlinieninzision werden v. a. im asiatischen Raum beschrieben [26]. Weitere Zugangswege, deren Ergebnisse erst weiter reevaluiert werden müssen, beinhalten retroaurikuläre, postaurikuläre und transorale Schnittführungen. Auch transorale Zugangswege in Kombination mit einer roboterassistierten Technik werden beschrieben, um eine bessere Übersicht zu erreichen [26].

#### Parapharyngeale Tumoren

Tumoren, die sich medial des tiefen Lappens der Gl. parotidea im parapharyngealen Raum befinden (Eisberg-Tumoren), können histologisch ebenfalls gutartige Speicheldrüsentumoren sein. Die häufigste Entität in dieser Lokalisation ist das pleomorphe Adenom mit 34 % [27]. Je nach Lage des Tumors sind unterschiedliche Techniken beschrieben. Bei einer Lage medial der Gl. parotidea sind sowohl die transzervikale/transparotideale Schnittführung als auch die transorale Schnittführung möglich [28]. Transorale Schnittführungen können mit einer roboterassistierten Operation kombiniert werden [29]. Bei dieser Technik kann eine Navigation (Image-Guidance) zu Hilfe genommen werden. Hierbei wird ein Schnitt in der pterygomandibulären Raphe gesetzt. Der M. palatoglossus und die Mm. constrictores werden durchtrennt. Auch der M. pterygoideus medialis kann zur Erweiterung der lateralen Achse gespalten werden. Durch das Endoskop kann der Parapharyngealraum überblickt und der Tumor disseziert werden. Reicht dies nicht aus, muss eine transzervikale Inzision zusätzlich angelegt werden [30]. Bei dieser Technik besteht allerdings ein erhöhtes Risiko einer Pharynxfistel. Aus diesem Grund wird die transzervikale Schnittführung auch heute noch am häufigsten verwendet. Hierfür wird 1–2 Querfinger unterhalb der Mandibula geschnitten. Nach Darstellung der Gefäße ist die Durchtrennung des M. digastricus venter posterior und die Resektion des Proc. styloideus notwendig, um den Dissektionsraum zu erweitern. Häufig können die Tumoren nach Darstellung gut in extrakapsulärer Technik stumpf herauspräpariert werden im Sinne einer ED oder sogar kapsulären Dissektion. Ein Fazialismonitoring ist empfohlen [31]. Für Tumoren, die von der Gl. parotidea in den parapharyngealen Raum wachsen, ist ein transparotidealer Zugang notwendig. Bei dieser Technik wird der komplette Fazialisfächer wie bei einer kompletten Parotidektomie dargestellt, und die Tumoranteile werden intraparotideal und im Parapharyngealraum in toto reseziert.

#### Rezidive von pleomorphen Adenomen

Die Inzidenz des ersten Rezidivs unterscheidet sich in mehreren Studien, abhängig von der Länge des Follow-up. Während sie in aktuellen Studien 1–2 % beträgt, erhöht sich diese bis auf 2 % nach 5 Jahren und auf 5–7 % nach 20 Jahren, unabhängig davon, welche Resektionstechnik verwendet wurde [32]. Generell kommen Rezidive v. a. bei Patientinnen (1,5:1) und durchschnittlich 7–10 Jahre nach Erstoperation vor [33]. Hierbei treten häufig (in 40–50 %) mehrere Rezidivknoten verteilt über die komplette anatomische Region (z. B. Parotisloge) auf. Intraoperativ finden sich neben den Knoten, die in der Magnetresonanztomographie (MRT) und in der Sonographie beschrieben sind, kleine 1 mm große Knoten im umliegenden Gewebe. Werden diese nicht komplett entfernt, kommt es zum nächsten Rezidiv [34].

Doch warum kommt es überhaupt zu Rezidiven von pleomorphen Adenomen? Die bekanntesten Ursachen liegen in den Besonderheiten, welche die pleomorphen Adenome aufweisen. Dies sind eine inkomplette Kapsel, Pseudopodien, Satellitentumoren und selten auch multifokale Tumoren [35, 36]. Pleomorphe Adenome werden histologisch nach Seifert in klassische/gemischte, hypozelluläre und hyperzelluläre Subtypen eingeteilt. Der hypozelluläre Subtyp (fragiler, mehr Satelliten, inkomplette Kapsel, jüngere Patientinnen und Patienten) und der hyperzelluläre Subtyp werden als deutlich rezidivanfälliger beschrieben als der klassische [3, 33, 37, 38]. Bei Rezidiven von pleomorphen Tumoren stellt sich zuerst die Frage, ob diese behandelt werden sollten. Dies ist ganz klar mit einem Ja zu beantworten. Die De-novo-Malignität bei nichtvorbehandelten pleomorphen Adenomen liegt bereits bei 1,1 %; bei primär resezierten reicht sie bis 24 % [3]. Die Frage nach dem Ausmaß und der Art der Behandlung ist jedoch weitaus weniger standardisiert. In der Regel handelt es sich um sehr individuelle Entscheidungsfindungen, wann und in welchem Ausmaß das Rezidiv operiert werden sollte. Wichtige Entscheidungskriterien sind neben der B‑Bild-Sonographie im Fall eines Rezidivs auch eine weitere Bildgebung mittels MRT sowie Informationen zum Voreingriff (Darstellung des N. facialis, pathologischer Bericht, ggf. Reevaluierung der Blöcke zum Ausschluss von Malignität). Außerdem ist der aktuelle House-Brackmann-Status als Surrogat für die aktuelle Fazialisfunktion ein wichtiger Bestandteil, der in die Entscheidungsfindung integriert wird. Letztendlich muss in die Therapieplanung insbesondere auch der/die Patienten/in mit einbezogen werden. Die Hauptindikation zur Rezidivoperation besteht auch darin, eine maligne Transformation auszuschließen, zu diagnostizieren und gleichzeitig zu therapieren. Eine Feinnadelpunktion wird nicht als adäquat dafür angesehen, eine Malignität auszuschließen [39].

In der Entscheidungsfindung müssen individuell Zahl und Verteilung der Rezidive sowie die aktuelle Fazialisfunktion miteingeschlossen werden. Die Schonung des N. facialis muss auch in jeder Revisionsoperation angestrebt werden und ist oberstes Gebot. Hierfür wird eine Kombination aus seiner Elektromyographie zum Nervenmonitoring und der Verwendung von Lupenbrillen empfohlen [40]. Die Wahrscheinlichkeit einer Nervenverletzung sollte mit der Patientin bzw. dem Patienten ausführlich diskutiert werden. Bei hoher Wahrscheinlichkeit einer (entstellenden) Nervenverletzung kann mit den Betroffenen individuell eine engmaschige Kontrolle („wait and scan/watch“) bzw. ein limitiertes operatives Vorgehen („node picking“) besprochen werden.

Bei der chirurgischen Therapie sollte die Hautnarbe der Voroperation wenn möglich exzidiert werden, die Fibrose um den N. facialis aber belassen werden [41]. Ist eine Nervendissektion unumgänglich, sollte eine retrograde Fazialisdissektion durchgeführt werden. Dabei sollte an einem Ort, der in der Voroperation nicht freigelegt wurde (z. B. distaler temporaler Ast), begonnen werden [42]. Intraoperativ sollte auch versucht werden, die kleinen, 1 mm großen Tumoren zu resezieren, um weitere Rezidive zu vermeiden bzw. die Wahrscheinlichkeit eines frühen Rezidivs zu vermeiden. Dennoch stellen gerade diese im Subkutangewebe gelegen Knoten die Hauptursache für erneute Rezidive dar. Daher muss auch neben der Resektion der Hautnarben eine Kompartmentresektion und die Resektion des N. facialis mit dem Patienten besprochen werden [43]. Eine adjuvante Radiotherapie (RT) kann bei bestimmten Befundkonstellationen (häufige und/oder multiple Rezidive, Knotengröße > 2 cm, myxoider Subtyp) nach Absprache mit dem Patienten durchgeführt werden [43, 44].

### Bösartige Speicheldrüsentumoren

#### Operationsplanung

Laut der 8. Edition der Leitlinie der Union for International Cancer Control (UICC) und dem aktuellen Update von 2022 sind 24 maligne histologische Subtypen beschrieben [1, 2]. Speicheldrüsenkarzinome sind selten und verursachen maximal 3–5 % aller Karzinome im Kopf-Hals-Bereich [45]. Die Inzidenz von Speicheldrüsenmalignomen beträgt etwa 1–2/100.000, abhängig von der Region [1, 45–47]. Primäre diagnostische Modalität ist der Ultraschall im Sinne einer B‑Bild-Sonographie. Sonographische Zeichen, die auf eine Malignität hindeuten können, sind unscharf begrenzte Ränder und eine Infiltration umliegender Strukturen [48]. Eine zusätzliche MRT kann bei Unsicherheit über die Dignität der Läsion durchgeführt werden [49]. Zum Staging ist eine Computertomographie (CT) von Hals und Thorax indiziert, laut den Leitlinien der American Society of Clinical Oncology (ASCO-Guidelines) sogar eine Positronenemissions(PET)-CT bis zur Mitte der Oberschenkel bei fortgeschrittenen und High-Grade-Tumoren. Zur Sicherung der Dignität ist eine Probenentnahme indiziert, entweder durch Feinnadel- oder durch Grobnadelbiopsie. Dies erleichtert einerseits die präoperative Planung, ist aber andererseits auch für die Aufklärung der Patientinnen und Patienten wichtig, da diese auch die Aufklärung über eine mögliche Notwendigkeit der Resektion und Rekonstruktion des N. facialis beinhaltet.

#### Malignome der Gl. parotidea

Der Goldstandard für maligne Speicheldrüsentumoren besteht aus einer kompletten Parotidektomie und einer ipsilateralen Neck-Dissection. Besteht nach einer extrakapsulären Dissektion oder superfiziellen Parotidektomie ein Malignitätsverdacht, so sollte laut der deutschen S3-Leitlinie „Speicheldrüsentumoren“ bei entsprechender pathologischer Expertise ein Schnellschnitt durchgeführt werden, um ggf. im gleichen Eingriff eine Komplettierung der Operation durchzuführen [5].

Dies ist v. a. nach erfolgter Nervendarstellung relevant, da in einem zweiten Eingriff die Rate an Fazialisparesen steigt und sich die Dissektion als schwieriger darstellt. Im Fall einer Infiltration des N. facialis ist eine radikale Parotidektomie indiziert. Dies gilt sowohl für eine bereits klinisch messbare (House-Brackmann, Elektromyographie) präoperative Fazialisparese als auch für eine intraoperative pathologische Infiltration des Nervs (z. B. bei adenoidzystischem Karzinom). Eine R1-Resektion am Nerv geht mit einem schlechteren onkologischen Outcome einher. Eine radikale Parotidektomie, wenn möglich und sinnvoll mit folgender dynamischer Nervenrekonstruktion, ist indiziert. Ist eine dynamische Nervenrekonstruktion u. a. aufgrund von präoperativ bestehender längerer Parese oder dem Nichtvorhandensein von Nervenenden zu Anastomosierung nicht möglich, dann kann eine statische Rekonstruktion durchgeführt werden [50].

Bei der Indikation zur Neck-Dissection muss zwischen einem klinisch und radiologisch positiven (cN+) und einem negativen (cN0) Halsstatus unterschieden werden. Wenn bildgebende Verfahren oder klinische Untersuchungen auf zervikale Lymphknotenmetastasen hinweisen, d. h. bei einem cN+-Hals, ist eine therapeutische Halsdissektion indiziert [5, 51, 52]. Nachdem die komplette bzw. radikale chirurgische Entfernung der Drüse der Goldstandard ist, ist auch die ipsilaterale Neck-Dissection im gleichen Eingriff problemlos durchführbar. Patienten ohne Hinweise auf Lymphknotenbefall hatten eine deutlich bessere Überlebensrate als Patienten mit pathologisch positiven Lymphknoten (mittlere Überlebensdauer: 100 vs. 59 Monate, *p* < 0,001) [53]. Bei einem cN0-Halsstatus ist die Indikation zur elektiven Neck-Dissection deutlich umstrittener. Viele Studien und Metaanalysen zeigen, dass das Risiko okkulter Lymphknotenmetastasen stark vom Tumorgrad, dem T‑Stadium und dem histologischen Subtyp abhängt. Faktoren, bei deren Vorhandensein eine elektive Neck-Dissection empfohlen wird, sind T3/4-Tumoren, High-Grade-Karzinome und adenoidzystische Karzinome [5, 49, 54, 55]. In einer Metaanalyse wurde eine gepoolte Rate okkulter Metastasen von etwa 21 % festgestellt; bei T3/T4-Tumoren lag sie bei etwa 36 %, und bei High-Grade-Tumoren bei etwa 34 % [56]. Lediglich bei kleinen Tumorstadien und Low-Grade-Tumoren kann auf eine Neck-Dissection bei geringem Risiko für okkulte Metastasen verzichtet werden. Die am häufigsten betroffenen Level bei Parotismalignomen sind Level II und III, Level I und V sind seltener betroffen. Die zusätzliche Morbidität der Level I und V wird jedoch als gering eingeschätzt, sodass auch diese entfernt werden können. Bei Patientinnen und Patienten mit Positivität verschiedener Level ist eine Neck-Dissection der Level I–V sinnvoll [57]. Bei hochgradigen oder T3/T4-Tumoren sollen die Level II–IV entfernt werden [49]. In einer retrospektiven Multizenterstudie aus den USA wurde gezeigt, dass die elektive Neck-Dissection keinen signifikanten Effekt auf das 3‑Jahres-Gesamtüberleben (*n* = 222, 3JÜ: 66,3 %) im Vergleich zu Patientinnen und Patienten ohne Neck-Dissection hatte (*n* = 76, 3JÜ: 63 %). Allerdings zeigte die elektive Neck-Dissection einen weiteren Benefit. Sie hat nicht nur therapeutischen Nutzen, sondern auch prognostischen [58]. Obwohl eine elektive Neck-Dissection bei adenoidzystischen Karzinomen generell empfohlen wird, ist dies teilweise differenziert zu betrachten. Zum einen metastasieren adenoidzystische Karzinome nur selten lymphogen (5–20 %). Dies trifft v. a. auf adenoidzystische Karzinome in der Gl. parotidea zu (10–15 %) [59]. Zum anderen metastasieren adenoidzystische Karzinome mit High-Grade-Transformationen in mindestens 50 % der Fälle in die Lymphknoten [60]. Bei Letzteren bzw. generell bei High-Grade-Tumoren ist immer eine elektive Neck-Dissection indiziert.

Verschiedene Studien haben den Wert der Sentinel-node-Biopsie bereits seit 1967 diskutiert [61]. Eine aktuelle Studie von Lombardi et al. schlägt vor, Level II als Sentinel-Level zu verwenden. In der Studie wurde das Level II intraoperativ zum Schnellschnitt geschickt und nur bei Positivität die restlichen Level III und IV komplettiert [62]. Wie bereits erwähnt, wiesen Patienten ohne Hinweise auf Lymphknotenbefall eine deutlich bessere Überlebensrate auf als Patienten mit pathologisch positiven Lymphknoten (mittlere Überlebensdauer: 100 vs. 59 Monate, *p* < 0,001) [53]. Auch das krankheitsspezifische Überleben (pN0: 85 %; pN1: 76,2 %; N2–3: 34 %; *p* > 0,001) [63] und das 10-Jahres-Überleben (pN0: 67 %, pN+: 24 %) unterscheiden sich signifikant, abhängig vom Halsstatus [64].

Eine radikale Parotidektomie bezeichnet die Entfernung der gesamten Ohrspeicheldrüse einschließlich der Mitresektion von Anteilen oder des gesamten N. facialis. Eine Indikation zur radialen Parotidektomie besteht bei Infiltration des N. facialis durch den Tumor (cT4a) [65, 66]. Dies ist unabhängig vom präoperativen Fazialisstatus. Eine bereits präoperativ eingeschränkte Fazialisfunktion ist jedoch zudem ein negativer prognostischer Faktor [67, 68]. Ziel der Operation sind freie Resektionsränder. Bei Infiltration des Tumors in Haut‑, Knochen- oder Schädelbasis ist eine erweiterte Resektion notwendig, um freie Resektionsränder zu erreichen [69]. Im Fall einer radikalen Parotidektomie ist eine Nervenrekonstruktion indiziert. Im Idealfall sollte diese im gleichen Eingriff erfolgen. Wenn möglich, sollte eine dynamische Fazialisrekonstruktion im Vergleich zu einer statischen Rekonstruktion für verbesserte funktionelle Ergebnisse durchgeführt werden [70]. Im Fall einer Hautresektion kann eine gestielte oder freie Lappenplastik zu Deckung notwendig sein. In einigen Fällen ist auch eine Resektion von Mastoid- oder temporalem Knochen notwendig [71]. Dies sollte präoperativ gründlich vorbereitet werden. Bei T3/4-Tumoren, High-Grade-Tumoren, perineuraler Invasion, positiven Rändern, pN+- und adenoidzystischen Karzinomen ist eine adjuvante Radiotherapie indiziert [5, 49].

#### Low-Grade-Karzinome der Gl. parotidea

In einer retrospektiven Fallserie von einer hochselektierten Patientenkohorte mit 44 Low-Grade‑, Low-Stage- und inferior lokalisierten Tumoren im Stadium T1–2 erhielten 14 Patientinnen und Patienten nach extrakapsulärer Dissektion keine Komplettierungsoperation (z. B. auf eigenen Wunsch). Die Nachsorge mit einer Dauer von 2,1 bis 8 Jahren (Mittelwert 4,5 Jahre) zeigte in diesem selektierten Patientengut von Low-Grade-Karzinomen (überwiegend Azinuszellkarzinome und Mukoepidermoidkarzinome, ein Karzinom ex pleomorphes Adenom, ein Basalzelladenokarzinom) eine bessere Fazialisfunktion direkt postoperativ, hinsichtlich der langfristigen Rate an Fazialisparesen bestand kein Unterschied. Auch die übrigen klinischen und onkologischen Outcomes waren alle vergleichbar [12]. Die Daten von 16 Patienten mit Zustand nach ED bei Low-Grade-Karzinomen im Stadium T1–2 wurden in einer weiteren Arbeit hinsichtlich der langfristigen Ergebnisse ausgewertet. Nach einem durchschnittlichen Follow-up waren alle Patienten tumor- und rezidivfrei. Dieses Vorgehen sollte jedoch nicht der Standard sein, sondern nur in der beschriebenen hochselektierten Patientengruppe mit guter Compliance und regelmäßigen Kontrollen (klinisch, Bildgebung mittels B‑Bild-Sonographie, z. B. 3‑monatlich, und MRT, z. B. jährlich) erfolgen [72]. In den aktuellen ASCO-Guidelines wurde die limitierte Resektion (partielle Parotidektomie) für derartige Tumoren als mögliche Therapie empfohlen (Recommendation 2.3) [49].

#### Gl. submandibularis und Gl. sublingualis

Im Gegensatz zur Gl. parotidea ist der Anteil an Malignität bei allen Tumoren in der Gl. submandibularis mit 50 % höher und in der Gl. sublingualis mit 80 % am höchsten [73]. Der Goldstandard bei beiden Lokalisationen ist die Entfernung der kompletten Drüse mit freien Resektionsrändern. Bei Infiltration in die umliegenden Strukturen wie N. lingualis, R. marginalis nervi facialis, Muskulatur, Knochen oder Haut sind diese Strukturen mitzuentfernen, um freie Resektionsränder zu erreichen. Bei der Resektion der Gl. sublingualis kann ein Defekt im Mundboden entstehen, der mittels einer gestielten oder freien Lappenplastik gedeckt werden muss. Dies ist präoperativ zu beachten. Die Indikation zur ipsilateralen Neck-Dissection kann analog zur Gl. parotidea gestellt werden. Eine elektive Neck-Dissection sollte allerdings die Level I–III umfassen [74]. Die Indikation zur adjuvanten Radiotherapie wird ebenfalls analog zur Gl. parotidea gestellt [5].

#### Kleine Speicheldrüsen

Während die großen Speicheldrüsen von einer Kapsel umgeben sind, fehlt diese bei den kleinen Speicheldrüsen. Die kleinen Speicheldrüsen sind in den kompletten oberen Schluck- und Atemwegen verteilt (insgesamt 450–1000), 90 % befinden sich allerdings in Mundhöhle und Oropharynx [75]. Geschätzt treten maligne Befunde der kleinen Speicheldrüsen in 0,4/100.000 Fällen auf. Tumoren der kleinen Speicheldrüsen sind in bis zu 50 % der Fälle maligne. Dies entspricht 10–15 % aller Speicheldrüsentumoren. In über 90 % der Fälle handelt es sich um polymorphe Adenokarzinome, adenoidzystische Karzinome (32–71 %) oder Mukoepidemoidkarzinome (15–38 %). Das Tumor-Staging erfolgt entsprechend der Klassifikation der Kopf-Hals-Plattenepithelkarzinome [5]. 90 % aller Malignome der kleinen Speicheldrüsen sind Low-Grade-Tumoren [76]. Bei malignen Tumoren der kleinen Speicheldrüsen ist eine operative Entfernung des Tumors mit freien Resektionsrändern der Goldstandard. Ein Resektionsrand von 1 cm wird empfohlen [77]. Jedoch gibt es hierfür keine gute Evidenz. Eine vergleichende Studie hat orale Plattenepithelkarzinome und orale Speicheldrüsenkarzinome verglichen. Beim oralen Plattenepithelkarzinom wird ein Resektionsabstand von 5 mm empfohlen. In dieser Studie zeigten orale Speicheldrüsenkarzinome trotz der häufigeren knappen oder sogar positiven Resektionsränder gegenüber oralen Plattenepithelkarzinomen weniger Lokalrezidive, was durch den hohen Anteil an Low-Grade-Karzinomen im Zusammenhang mit der durchgeführten adjuvant RT begründbar ist [29]. Die Lage und die Ausdehnung des Tumors gepaart mit der fehlenden Tumorkapsel erschweren die Resektion im Gesunden im Vergleich zu den genannten großen Speicheldrüsen. Eine Neck-Dissection bei pN+-Hals ist indiziert, bei oraler oder oropharyngealer Lokalisation, bei T3/4-Tumoren und bei High-Grade-Tumoren indiziert, bei mittelliniennahen Tumoren der Mundhöhle bzw. des Oropharynx auch beidseitig. Bei cN+-Status von Tumoren der Mundhöhle sollten die Level I–IV, bei oropharyngealen Tumoren die Level II–V ausgeräumt werden [77]. Ein besonderes Augenmerk sollte auch auf Lymphknoten außerhalb der genannten Level gelegt werden, z. B. retropharyngeale oder mediastinale Lymphknoten. Bei Tumoren im oropharyngealen Bereich kann durch die Tumorresektion ein durchgreifender Defekt entstehen, bei dem eine gestielte oder freie Lappenplastik zur Deckung notwendig sein kann. Die Indikation zur adjuvanten Radiotherapie wird analog zur Gl. parotidea gestellt.

## Teil 2: Update der Chirurgie bei obstruktiven und inflammatorischen Speicheldrüsenkrankheiten

### Entwicklung der minimal invasiven Therapieverfahren und State of the Art

Bis vor 35 Jahren wurden mehr bei mehr als 50 % aller Patienten mit obstruktiven/inflammatorischen Erkrankungen der großen Kopfspeicheldrüsen die Drüsen operativ entfernt. Seitdem wurde die Drüsenresektion sukzessive verlassen und durch minimal invasive Verfahren ersetzt, welche seitdem sukzessive implementiert wurden. Die extrakorporale Stoßwellenlithotripsie (ESWL) [78] wurde seit 1989 und die transorale Gangchirurgie (TGS) [79] seit Beginn der 1990er-Jahre durchgeführt. 1990 wurde die fiberoptische Inspektion der Speicheldrüsengänge [80] und die fiberoptisch kontrollierte intraduktale Laser-Lithotripsie von Speichelsteinen erstmals beschrieben [81]. Die therapeutische bzw. interventionelle Sialendoskopie gewann jedoch erst zu Beginn der 2000er-Jahre, nach Entwicklung von geeigneten Instrumenten [82, 83], mehr und mehr an Bedeutung und ist zunehmend zur dominanten Modalität insbesondere bei obstruktiven Speichelgangkrankheiten geworden. In verschiedenen Reviews und Metaanalysen wurden für unterschiedliche sialendoskopisch kontrollierte Eingriffe Erfolgsraten zwischen 76 und 86 % beschrieben [73, 84, 85]. Schwarz et al. publizierten einen weiteren Review, welcher die Ergebnisse nach therapeutischer oder interventioneller Sialendoskopie bei Kindern und Jugendlichen untersuchte (17 Artikel mit 323 Patienten, 424 Drüsen). In 68,9 % der Fälle wurde eine chronische rezidivierende juvenile Parotitis, in 14,7 % eine Sialolithiasis, in 7,2 % eine Gangstenose und in 2,1 % eine Sialadenitis anderer Ursache therapiert. Das rezidivfreie Outcome lag bei 85,5 % [86].

Sialendoskopisch assistierte operative Verfahren wie die kombinierte endoskopisch-transkutane Chirurgie, erstmals 2002 beschrieben [87], und die endoskopisch assistierte transorale Chirurgie, seit Anfang der 2000er-Jahre beschrieben für verschiedene Indikationen [88–90], erweiterten die Möglichkeiten der Sialendoskopie.

Auch nach der Entwicklung von sialendoskopiebasierten Verfahren zeigte eine Auswertung von mehreren Speicheldrüsenzentren, welche nahezu 5000 Patienten einschloss, dass die höchsten Erfolgsraten nach einer kombinierten, multimodalen Therapie erreicht wurden, die Speicheldrüsen mussten in nur 2,9 % entfernt werden [91]. Die zunehmende Rolle der sialendoskopisch kontrollierten Therapieverfahren spiegelt sich in den ersten publizierten Therapiealgorithmen wider [92–97].

Zwischen 2010 und 2015 reiften sowohl das Equipment als auch das Verfahren der intraduktalen Stoßwellenlithotripsie (ISWL) mehr und mehr aus, was zum einen durch die Entwicklung geeigneter Instrumente und zum anderen durch die Entwicklung geeigneter Lithotripter möglich wurde. Mithilfe dieser Innovationen war es möglich, auch große, impaktierte Steine mit schwieriger Lokalisation, wie sie häufig bei der multiplen Sialolithiasis vorliegen, effektiv zu therapieren. Insbesondere während der letzten 10 Jahre sind die Erfolgsraten der ISWL deutlich gestiegen und liegen zzt. bei deutlich über 90 %, teilweise auch über 95 %. Die ISWL wird derzeit mittels pneumatischer [98–100] und mittels der Laser-Lithotripsie [101, 102] durchgeführt, wobei hinsichtlich der laserbasierten Technik aktuell Holmium-YAG-Laser-Verfahren dominieren [101, 102]. Zeitgleich wurde von mehreren Arbeitsgruppen über die transorale roboterassistierte Extraktion von tief liegenden großen Steinen der Gl. submandibularis berichtet [103–105], auch in Kombination mit der Sialendoskopie [106, 107]. Für diese Verfahren wurden ebenfalls Erfolgsraten von über 90 % publiziert.

Daneben wurden in den letzten Jahren auch über Verfahren berichtet, welche die Sialendoskopie einschlossen bzw. als sialendoskopisch assistierte Operationen mit simultaner Bildgebung durchgeführt wurden. So stellte die simultane Anwendung der Sialendoskopie mit dem Ultraschall insbesondere bei der Therapie von Steinen und Stenosen einen Fortschritt bei komplexen und therapieresistenten Situationen dar [108, 109]. Die Ultraschall- oder CT-Navigation stellen Modifikationen der sialendoskopisch assistierten transkutanen Operation dar, wodurch das Indikationsspektrum dieses Verfahrens, insbesondere für Steine mit schwieriger Lokalisation, erweitert werden konnte [110–114].

### Sialolithiasis

#### (Interventionelle) Sialendoskopie

Die alleinige interventionelle Sialendoskopie mit Extraktion von Steinen mittels verschiedener Instrumente wie Körbchen oder Zängelchen ist zwar das Verfahren erster Wahl, gelingt jedoch nur bei kleinen und mobilen Steinen, was in 5–10 % der Fall ist. Weitere 5–10 % der Steine können mittels Minibohrer manuell mechanisch fragmentiert und die Fragmente dann endoskopisch entfernt werden (eigene Daten, nicht publiziert, und [115–117]). Mehrere Arbeitsgruppen zeigten, dass die Erfolgsraten mittels einer Kombination von interventioneller Sialendoskopie und TGS, ESWL oder endoskopisch assistierter transkutaner Chirurgie insbesondere bei Steinen der Gl. parotidea um bis zu 35 % gesteigert werden konnten [117–119].

#### Endoskopisch kontrollierte intraduktale Stoßwellenlithotripsie

**Abb. 5:**
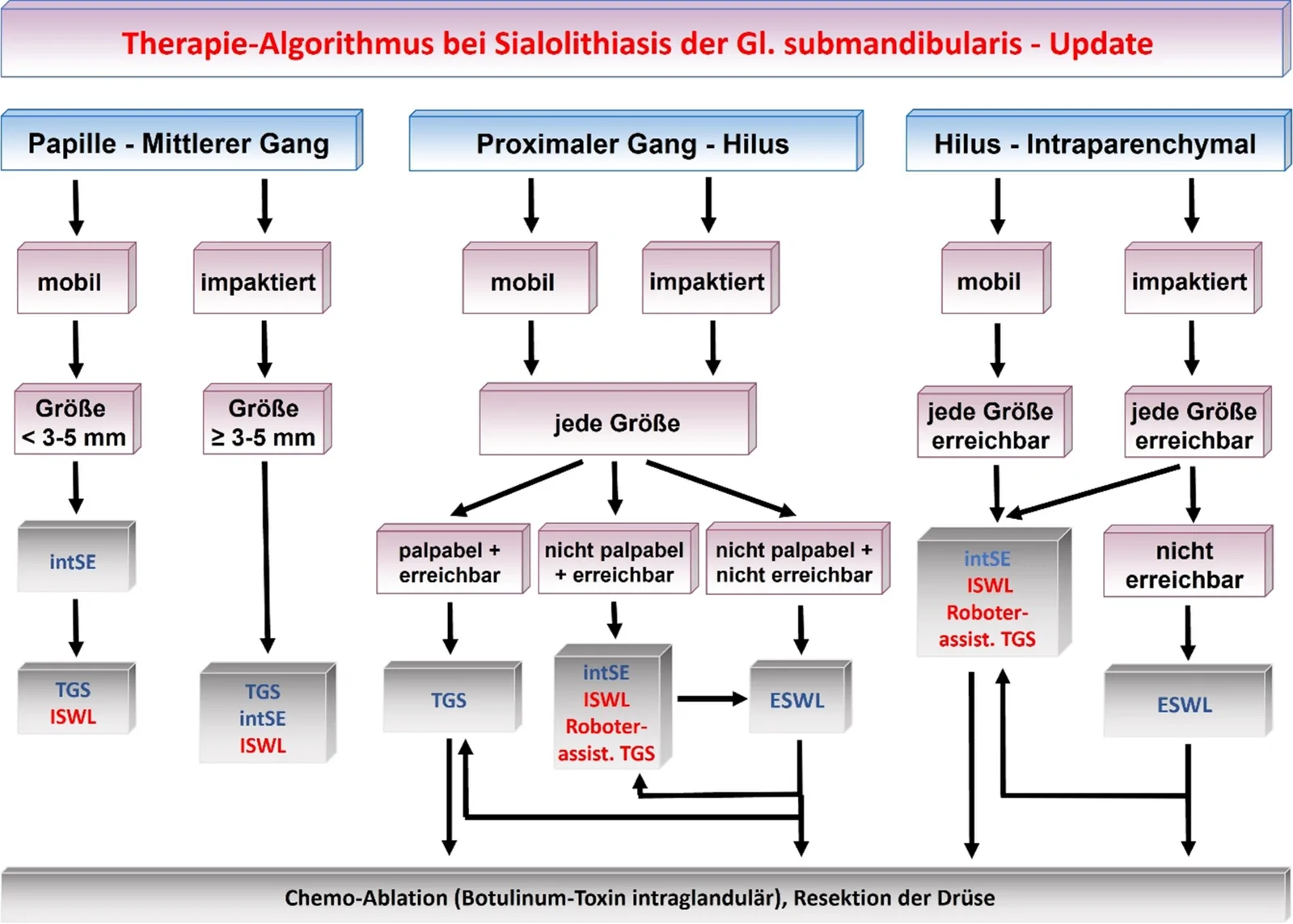
Algorithmus für die Therapie der Sialolithiasis der Gl. submandibularis. *assist. *assistiert, *ESWL *extrakorporale Stoßwellenlithotripsie, *intSE* interventionelle Sialendoskopie, *ISWL *intraduktale Stoßwellenlithotripsie,* TGS* transorale Gangchirurgie

Die Technik der intraduktalen Stoßwellenlithotripsie (ISWL) ist in den zurückliegenden 10 Jahren deutlich verfeinert worden und führte zu deutlich besseren Ergebnissen insbesondere bei größeren impaktierten Steinen. In der Erlanger Klinik werden derzeit 30–35 % aller Fälle mit Sialolithiasis in der Gl. submandibularis und 70–80 % mit Steinen in der Gl. parotidea mittels ISWL therapiert (eigene Daten, nicht publiziert), was den Stellenwert dieser Technik in dem aktuellen Therapiealgorithmus unterstreicht (Abb. 5 und 6; [120]).

**Abb. 6:**
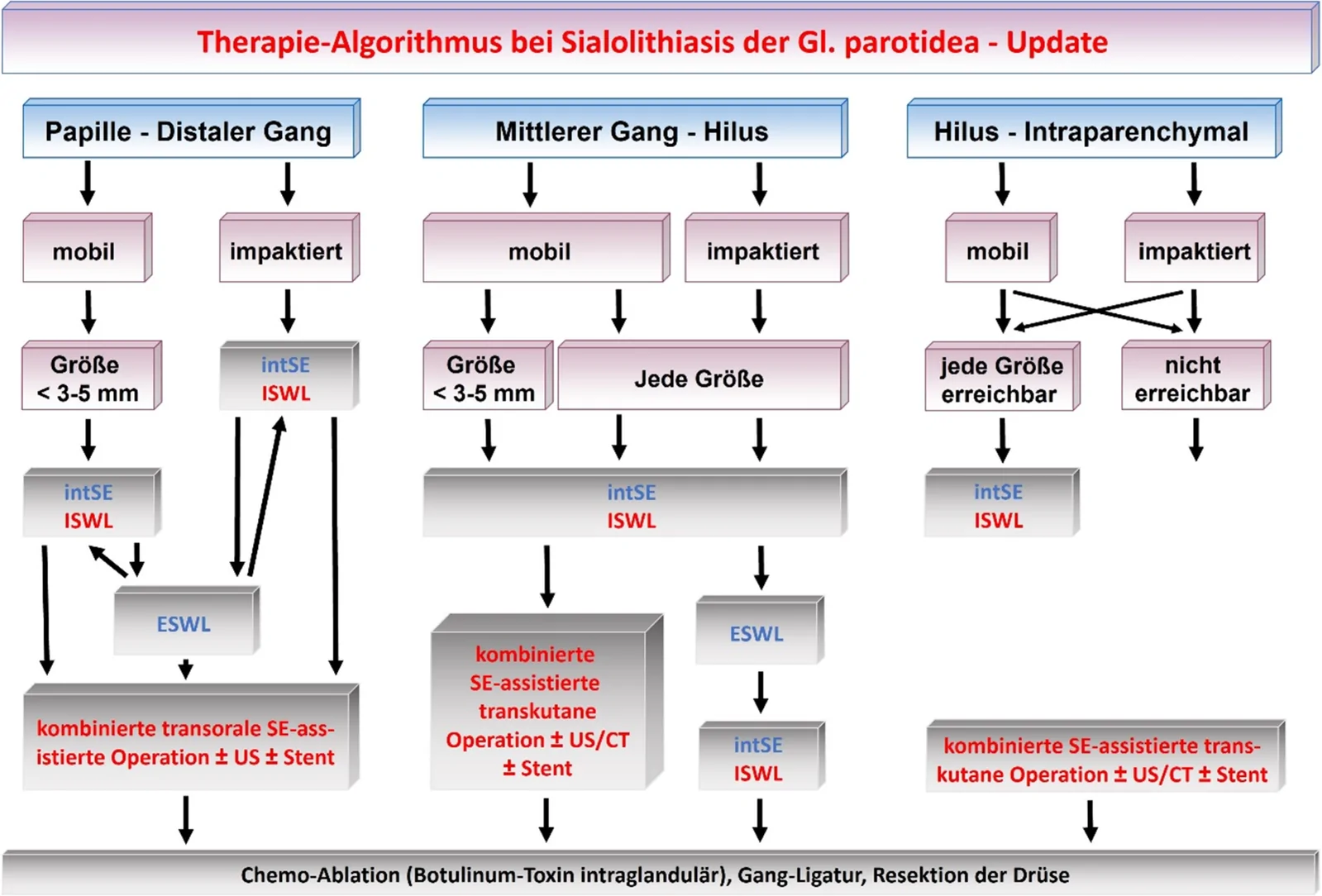
Algorithmus für die Therapie der Sialolithiasis der Gl. Parotidea. *assist. *assistiert, *CT* Computertomographie, *ESWL *extrakorporale Stoßwellenlithotripsie, *intSE* interventionelle Sialendoskopie, *ISWL *intraduktale Stoßwellenlithotripsie,* SE* Sialendoskopie, *TGS* transorale Gangchirurgie, *US* Ultraschall

Bei der pneumatischen Lithotripsie werden Steine mittels ballistischer/kinetischer Energie, welche über eine Metallsonde übertragen wird, fragmentiert. In den bisher publizierten Arbeiten wurde über sehr gute Erfolgsraten berichtet. Die aktuelle Literatur zeigt, dass die Steinfragmentierung in über 90 % bis zu 100 % der Fälle möglich ist, über 90 % bis zu 98 % der Patienten wurden steinfrei, in bis zu 100 % der Fälle wurden die Patienten beschwerdefrei, und die Drüse konnte erhalten bleiben [98–100, 121–123]. Für die pneumatische Lithotripsie steht z. B. der SialoLither™ (Fa. HidroMed, Ankara, Türkei) zur Verfügung, welcher auch eine Zulassung für Speichelsteine hat. Das Gerät verfügt derzeit über eine Zertifizierung der Europäischen Kommission (EG) gemäß der Medizinprodukte-Verordnung für die Behandlung von Speichelsteinen.

Bei der Laser-Lithotripsie werden Steine mittels lichtthermischen Energiepulsen, welche über eine Faser übertragen werden, fragmentiert. Die Laser-Lithotripsie wird aktuell nahezu ausschließlich mit einem Holmium-YAG-Laser (2100 nm) durchgeführt [101, 102]. In einem systematischen Review wurden die Ergebnisse nach laserassistierter Lithotripsie untersucht. 16 Studien mit 379 therapierten Patienten wurden in die Auswertung eingeschlossen. In 11 Studien wurde ein Holmium-YAG-Laser verwendet. Die Erfolgsrate betrug 71–100 %, der Erhalt der Drüse gelang in 97 % der Fälle [102]. In einer Studie unserer Arbeitsgruppe konnten alle Steine komplett fragmentiert werden, 95,9 % aller Patenten waren steinfrei, alle Patienten waren beschwerdefrei, und in allen Fällen konnte die Drüse erhalten werden [101].

In 2 Studien wurden die Ergebnisse nach pneumatischer und Laser-Lithotripsie verglichen. Jeweils über 90 % aller Fälle waren stein- und beschwerdefrei, die Speicheldrüsen konnten in allen Fällen erhalten werden, eine signifikante Verbesserung der Lebensqualität postinterventionell wurde nach beiden Verfahren beschrieben. Die Op.-Dauer war allerdings nach pneumatischer Lithotripsie signifikant geringer. Keine Unterschiede ergaben sich hinsichtlich der Rate relevanter Major-Komplikationen, welche jeweils unter 5 % lagen [121, 123].

Die Kombination der ISWL mit anderen Therapiemodalitäten erwies sich insbesondere in schwierigen Fällen, v. a. bei drüsennaher und/oder multipler Sialolithiasis, als entscheidend für den Therapieerfolg. In Fällen mit schwieriger Sialolithiasis waren nach Kombination der TGS mit der ISWL in der Gl. submandibularis 97 % aller Patienten [124] bzw. nach Kombination der ESWL und der ISWL in beiden Drüsen über 90 % der Patienten stein- und beschwerdefrei [125].

In einem systematischen Review, in welchem die Therapie der Sialolithiasis der Gl. parotidea untersucht wurde, wurden 1285 Fälle nach minimal invasiver Therapie in 13 Studien ausgewertet. Neben der Sialendoskopie, der ISWL und ESWL wurden weitere operative Manipulationen am Gangsystem inklusive endoskopisch assistierter Verfahren eingeschlossen. Die Erfolgsrate zeigte eine große Spannweite und betrug 71,4–100 %, durchschnittlich 88,7 %. Insbesondere spielte die Lokalisation der Steine eine Rolle, die Erfolgsraten nahmen von distal nach intraparenchymal ab von 100 bis 80 %. Erwähnenswert war auch, dass zwar über Minor-, jedoch über keine Major-Komplikationen berichtet wurde, was unterstreicht, dass die sialendoskopisch kontrollierte minimal invasive Therapie der Sialolithiasis effektiv und sicher ist [73]. Die ISWL wird aktuell nur in spezialisierten Zentren angeboten und ist nicht flächendeckend national und international verfügbar.

Die Wertigkeit der Sialendoskopie in der Therapie der Sialolithiasis bei Kindern und Jugendlichen unter 18 Jahren, welche insgesamt etwa 3 % aller Fälle mit Sialolithiasis ausmachen, wurde in einem systematischen Review und einer Metaanalyse untersucht. 13 Studien mit 133 Patienten wurden darin eingeschlossen. Die gewichtete und gepoolte Effektivität bzw. Erfolgsrate betrug 95,5 % (95 %-KI: 89,8–99,3 %). Die gewichtete gepoolte Sicherheit der sialendoskopisch kontrollierten Verfahren betrug 97,2 % (95 %-KI: 91,8–100 %), es wurde über keine Major-Komplikationen in den eingeschlossenen Studien berichtet. Die Therapie erfolgte bevorzugt in Allgemeinanästhesie, eine Therapie in Lokalanästhesie ab 8 Jahren. Die Autoren schlossen aus diesen Ergebnissen, dass die Sialendoskopie als First-Line-Verfahren angesehen, jedoch auch als Teil einer kombinierten Therapie angewendet werden sollte [126].

#### Transorale Gangchirurgie und Modifikationen

Neben der konventionellen transoralen Gangchirurgie (TGS) wurde die sialendoskopisch assistierte TGS in der Gl. submandibularis als eine weitere Modifikation publiziert. Hierbei ist das Ziel, den Stein mittels Sialendoskopie zu markieren, den Gang sialendoskopisch navigiert direkt über dem Stein zu eröffnen und nach Steinextraktion den Gang wieder zu verschließen, entweder mit oder ohne Stentimplantation. Da das Verfahren mit einem höheren instrumentellen und personellen Aufwand verbunden ist, erfolgte die Operation daher überwiegend in Allgemeinanästhesie [88, 127]. In einer Metaanalyse, welche 91 Studien mit insgesamt 8218 Patienten umfasste, wurden auch die gepoolten Erfolgsraten nach sialendoskopisch assistierten Verfahren verglichen. Die Erfolgsraten nach transoraler endoskopisch assistierter Steinentfernung der Gl. submandibularis betrugen hierbei 86,3 % (95 %-KI: 77,4–92,0; 8 Studien eingeschlossen) [85]. Sie sind damit vergleichbar mit den Erfolgsraten nach konventioneller TGS [128, 129]. Eine weitere Modifikation der TGS stellt die Durchführung unter mikroskopischer Kontrolle dar. Publiziert wurde sie für die Therapie von größeren Steinen, die Erfolgsraten hinsichtlich der Steinextraktion lagen auch bei dieser Variante bei über 90 %. Die roboterassistierte TGS erlaubt die Steinextraktion unter höherer visueller Auflösung und mit größerer mechanischer Flexibilität. Erfolgsraten von 86,5–100 % hinsichtlich einer kompletten Steinextraktion und 94–100 % hinsichtlich des Erhalts der Drüse wurden berichtet [103, 105, 130]. Allerdings ist die roboterassistierte TGS nur in Allgemeinanästhesie durchzuführen, aufwendig und teuer; sie zeigte eine deutlich reduzierte Kosteneffizienz im Vergleich zu den konventionellen Techniken mit Kosten von durchschnittlich fast 17.000 US-Dollar [105]. In einem systematischen Review wurden Erfolgsraten nach roboterassistierter TGS, welche mit oder ohne Kombination mit einer Sialendoskopie erfolgte, von 90,9–100 % beschrieben. In 28,3 % der Fälle trat eine transiente Läsion des N. lingualis auf, in keinem Fall eine permanente. Die Dauer des Eingriffs betrug durchschnittlich 91 min [131]. In einer weiteren Arbeit wurde die mikroskopisch kontrollierte TGS mit der roboterassistierten TGS verglichen. Die Erfolgsraten für die Steinextraktion waren für beide Verfahren vergleichbar hoch mit über 90 %, auch die Komplikationsraten waren vergleichbar. Die Op.-Dauer war jedoch bei der roboterassistierten TGS signifikant länger [132].

In einer Metaanalyse, welche 23 Studien bzw. 2520 therapierte Patienten einschloss, wurden die Ergebnisse nach unterschiedlichen Variationen der TGS und roboterassistierter TGS verglichen. Die Erfolgsraten waren 95,2 % (95 %-KI: 91,6–97,9) für die endoskopisch assistierte TGS sowie 90,3 % (95 %-KI: 83,08–95,45) für die konventionelle TGS. Sie betrugen 92,6 % (95 %-KI: 89,3–95,4) für alle Modifikationen der TGS zusammen. Für die roboterassistierte TGS wurde eine etwas bessere Erfolgsrate von 95,7 % (95 %-KI: 90,9–99,0) kalkuliert. Transiente Läsionen des N. lingualis wurden häufiger nach roboterassistierter TGS als nach TGS beschrieben (15,8 vs. 8,1 %), ebenso wie Komplikationen insgesamt (15,8 vs. 13,1 %). Die Autoren folgerten aus diesen Ergebnissen, dass die roboterassistierte TGS zwar aufgrund der exzellenten Steinextraktionsraten eine Therapieoption darstellt, jedoch aufgrund der hohen Kosten nur selektiv indiziert werden sollte [133].

Operative Verfahren der TGS bei Steinen der Gl. parotidea sind nicht indiziert, da die Steine i. d. R. mittels interventioneller Sialendoskopie bzw. ISWL therapierbar sind. In den wenigen Ausnahmefällen, in welchen eine endoskopisch kontrollierte Therapie allein nicht möglich oder sinnvoll ist, kann die transorale retropapilläre Steinextraktion die Therapie der Wahl darstellen. Hierbei wird die Papille nicht tangiert, sondern der Gang retropapillär dargestellt, eröffnet und anschließend der Stein extrahiert, meist mit Implantation eines Stents. 96–100 % aller Patienten wurden nach diesem Verfahren steinfrei, 83–100 % beschwerdefrei, die Drüsen konnten in allen Fällen erhalten werden [90, 96, 134–136].

In eine Metaanalyse, welche 91 Studien mit insgesamt 8218 Patienten umfasste, wurden auch 8 Studien eingeschlossen, aus welchen eine gepoolte Erfolgsrate von 86,3 % (95 %-KI: 77,4–92,0) nach transoraler endoskopisch assistierter Steinextraktion sowohl bei der Gl. submandibularis als auch der Gl. parotidea errechnet wurde [85].

#### Sialendoskopisch assistierte transkutane Chirurgie

Die sialendoskopisch assistierte transkutane Operation wurde nahezu ausschließlich für die Therapie der Sialolithiasis der Gl. parotidea etabliert. Hierbei wird der Stein mittels des Sialendoskops dargestellt, mittels Diaphanoskopie markiert und über einen transkutanen Zugang mit Navigation per gleichzeitiger Diaphanoskopie über eine Eröffnung des Gangsystems entfernt. In mehr als 20 Publikationen wurde über die Erfahrungen und Ergebnisse mit sialendoskopisch assistierten transkutanen Operationen der Gl. parotidea berichtet. 75–100 % aller Patienten wurden steinfrei oder beschwerdefrei, in 83,3–100 % aller Fälle konnte die Drüse erhalten werden [87, 88, 96, 111, 134, 136, 137]. Eine Metaanalyse wertete die Ergebnisse von 10 der ersten Publikationen mit insgesamt 184 therapierten Patienten aus. Die gewichteten gepoolten Erfolgsraten waren 99 % (95 %-KI: 97–100 %) für die Steinentfernung, 97 % (95 %-KI: 93–99 %) für die Verbesserung der Symptome, und 100 % (95 %-KI: 99–100 %) hinsichtlich des Drüsenerhalts. Die gewichtete gepoolte Komplikationsrate betrug 6 % (95 %-KI: 1–15 %), wobei auch Minor-Komplikationen (z. B. spontaner Stentabgang) mit eingerechnet wurden. Die Autoren folgerten daraus, dass die kombinierte endoskopisch-transkutane Operation ein sicheres und effektives Verfahren für Steine darstellt, welche mittels transoraler und endoskopischer Verfahren nicht therapierbar sind [138].

Modifizierte Verfahren, welche eine Steinextraktion auch ohne direkte sialendoskopische Steindarstellung infolge einer ungünstigen Lokalisation und/oder Begleitpathologie (z. B. Gangstenose) ermöglichen, wurden als Erweiterung der sialendoskopisch assistierten transkutanen Operation entwickelt. Steine, welche nicht mehr mit dem Sialendoskop direkt erreichbar waren, konnten unter Zuhilfenahme der Ultraschallnavigation [108, 110, 111] oder CT-Navigation [112–114] lokalisiert und entfernt werden. Alle auf diese Weise behandelten Patienten wurden steinfrei und beschwerdefrei. Diese Modifikationen sind erfolgreich durchgeführt worden, sind aber mit einem erheblichen personellen und apparativen Aufwand verbunden und daher nur in selektierten Fällen indiziert. Die kombinierten Verfahren stellen eine sinnvolle und effektive Erweiterung im Spektrum der operativen Techniken für die Sialolithiasis insbesondere der Gl. parotidea dar. Für alle Variationen der kombinierten transoralen oder transkutanen Chirurgie der Gl. parotidea wurde in einer Metaanalyse, welche insgesamt 91 Studien mit insgesamt 8218 Patienten umfasste und für diese Fragestellung 14 Studien einschloss, eine gepoolte Erfolgsrate von 78,2 % (95 %-KI: 66,7–86,5) kalkuliert [85].

Für die Gl. submandibularis wurde diese Operation in nur einer Publikation beschrieben. Es wurden bei 8 Patienten Steine, welche sehr tief posthilär bis intraparenchymal lokalisiert und mittels interventioneller Sialendoskopie oder TGS nicht therapierbar waren, mittels dieser kombinierten Operation mit einer Erfolgsrate von 100 % entfernt [139]. Allerdings standen offenbar Verfahren wie die ISWL oder ESWL nicht zur Verfügung. Vor diesem Hintergrund stellt diese Operationsmethode für die Gl. submandibularis keine relevante Therapieoption dar.

Insgesamt demonstrieren die für die Sialolithiasis dargestellten unterschiedlichen Operationsverfahren klar, dass kein einziges Verfahren allein den maximalen Therapieerfolg garantiert. Die publizierten Arbeiten [92–97] und aktualisierten Therapiealgorithmen [120] zeigen vielmehr, dass ein multimodales Therapiekonzept, welches auch schwierige Fälle der Sialolithiasis mit abdeckt, mit den höchsten Erfolgsraten verbunden ist. Das neueste Update zeigt aber auch, dass nach Implementierung der ISWL die sialendoskopisch kontrollierten Verfahren im aktuellen Therapiekonzept einen signifikanten Stellenwert haben. Unter Einbeziehung der aktuellen publizierten Daten in der Literatur ergeben sich aktualisierte Therapiealgorithmen für die Therapie der Sialolithiasis der Gl. submandibularis (Abb. 5) und der Gl. parotidea (Abb. 6), welche eine Modifikation des letzten Updates [120] erforderlich machten.

Das Vorliegen einer multiplen Sialolithiasis bedeutet i. d. R., dass neben einer multimodalen Therapie auch mehr als ein Therapiezyklus notwendig sein kann, was mit dem Patienten vorher kommuniziert werden sollte.

### Speichelgangstenosen

#### Interventionelle Sialendoskopie

Die interventionelle Sialendoskopie ist bei der Therapie von Speichelgangstenosen erste Wahl. Die instrumentelle Eröffnung hat Erfolgsraten von bis zu 90–95 %, eine Spülung mit kochsalz- oder kortisonhaltiger Spüllösung ist nahezu immer, die Stenteinlage in der Mehrzahl der Fälle indiziert [140]. In einer Studie wurde gezeigt, dass eine mehrwöchige Gabe von Kortison p.o. die Ergebnisse v. a. in schwierigen Fällen verbesserte und die Rezidivraten senkte [141]. Die simultane Anwendung von Ultraschall und Sialendoskopie (ultraschallnavigierte Sialendoskopie) kann eine erfolgreiche Therapie insbesondere bei kompletten Gangstenosen ermöglichen [109]. Eine systematische Analyse von Ergebnissen nach Therapie einer Speichelgangstenose zeigte, dass die die interventionelle Sialendoskopie bei Stenosen der Gl. parotidea in 60 % der Fälle das entscheidende Verfahren war [89, 142]. Durch die therapeutische und interventionelle Sialendoskopie konnten über 80 % aller Fälle erfolgreich therapiert werden [143]. Dagegen war dies in nur 27 % der Gangstenosen der Gl. submandibularis der Fall [143]. Sialendoskopisch kontrollierte Verfahren sind daher bei der Therapie von Speichelgangstenosen der Gl. parotidea von wesentlich größerer Bedeutung.

#### Sialendoskopisch assistierte transorale Chirurgie

Neben der interventionellen Sialendoskopie ist die TGS bei Stenosen der Gl. submandibularis erste Wahl. Die TGS ist aufgrund der operativen Erreichbarkeit des Gangsystems bei der Gl. submandibularis ein sehr effektives Verfahren und stellte daher in etwa 60 % aller Fälle die erfolgreiche Therapie dar [143].

Im Vergleich dazu sind bei der Gl. parotidea operative Manipulationen wie die TGS als Routinemethode kontraindiziert, insbesondere im Papillenbereich. Eingriffe wie die (erweiterte) Papillotomie können zu kompletten Stenosen führen, welche schwierig zu behandeln sind. Die retropapilläre Inzision mit Bildung eines Neoostiums oder die Gangresektion mit Gangreinsertion sind jedoch bei kompletten und/oder therapieresistenten Stenosen eine gute Therapiealternative und können mit Erfolgsraten von über 80 bis zu 90 % durchgeführt werden [89, 142]. Die Gangresektion mit Reinsertion wurde in der Literatur für die Therapie bei Megaduktus auch unter der Bezeichnung „Pull-through-Technik“ publiziert, mit komplettem Erfolg in 60 % und partiellem Erfolg in 40 % der Fälle [144]. Vor Kurzem wurde eine Modifikation dieser Technik vorgestellt, bei welcher zusätzlich eine operative Reduktion des Ganglumen zur Therapie eines Megaduktus erfolgte [145].

#### Sialendoskopisch assistierte transkutane Chirurgie

Obwohl diese Operation bei der Sialolithiasis sehr erfolgreich durchgeführt wird, hat sich dieses Verfahren wegen der schlechten Ergebnisse bisher nicht für die Therapie von kompletten, persistierenden Gangstenosen durchgesetzt. Oft muss hierbei eine Gangrekonstruktion mit Venenpatch oder -interponat durchgeführt werden. Eine hohe inflammatorische Komponente bei simultaner insuffizienter Drüsenfunktion sind die Hauptursachen für die schlechten Resultate. In mehreren Publikationen wurde diese Technik zur Therapie von Stenosen daher auch lediglich für Einzelfälle beschrieben [88, 111, 137].

Eine detaillierte Analyse der minimal invasiven Therapieverfahren und eine Auswertung der Ergebnisse für die Gl. submandibularis [143] und Gl. parotidea [142, 146] wurde von unserer Arbeitsgruppe publiziert. Die Ergebnisse zeigen, dass sialendoskopiebasierte Verfahren bei der Therapie von Gangstenosen in über 90 % aller Fälle eine entscheidende Rolle spielen. Bei Gangstenosen der Gl. submandibularis ist jedoch die TGS häufig die effektivere primäre Therapieform. Bei schwierigen und/oder therapieresistenten Speichelgangstenosen kann eine multimodale und/oder kombinierte Therapie erforderlich sein.

**Abb. 7:**
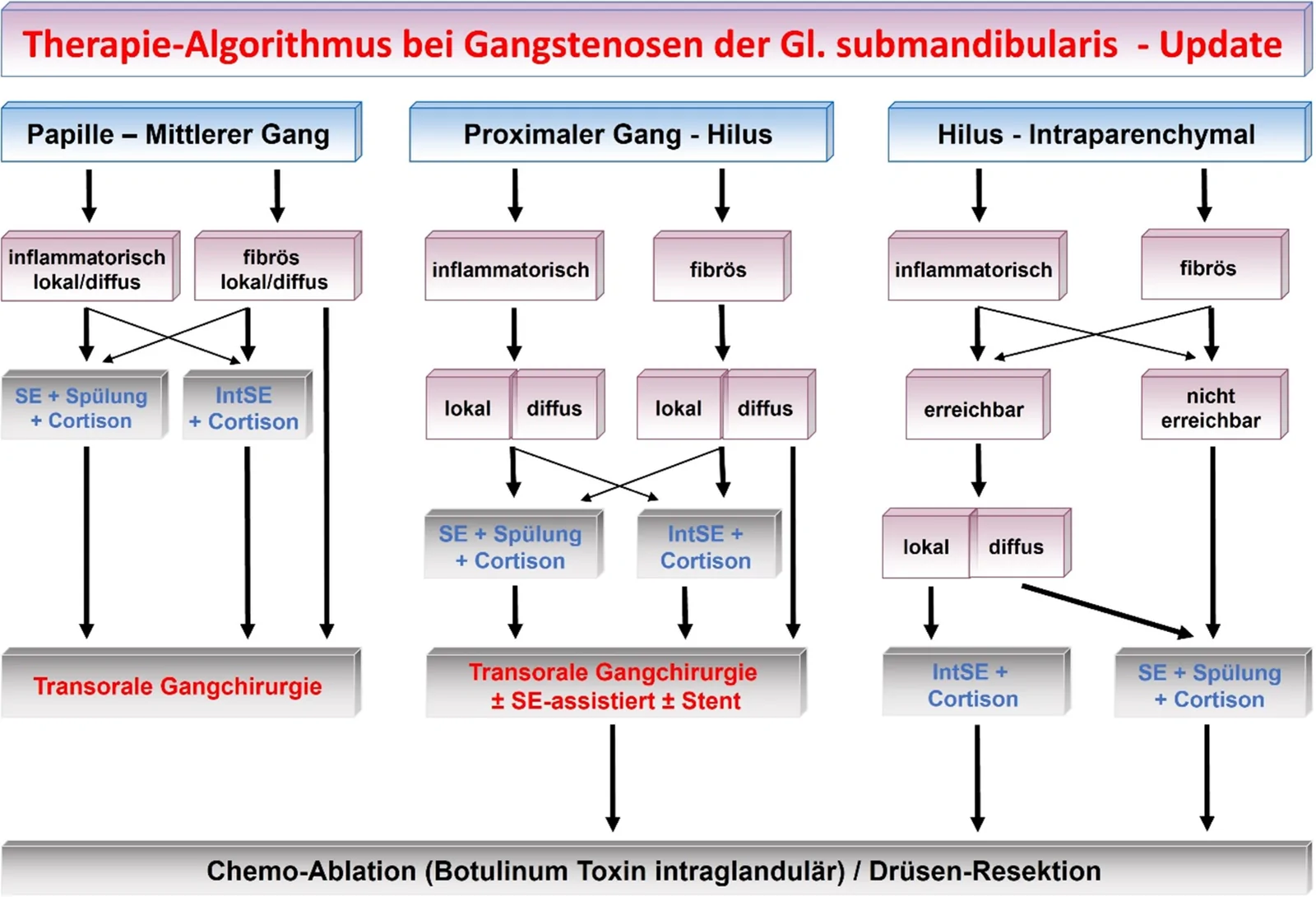
Algorithmus für die Therapie von Gangstenosen der Gl. Submandibularis. *IntSE* interventionelle Sialendoskopie, *SE* Sialendoskopie

**Abb. 8:**
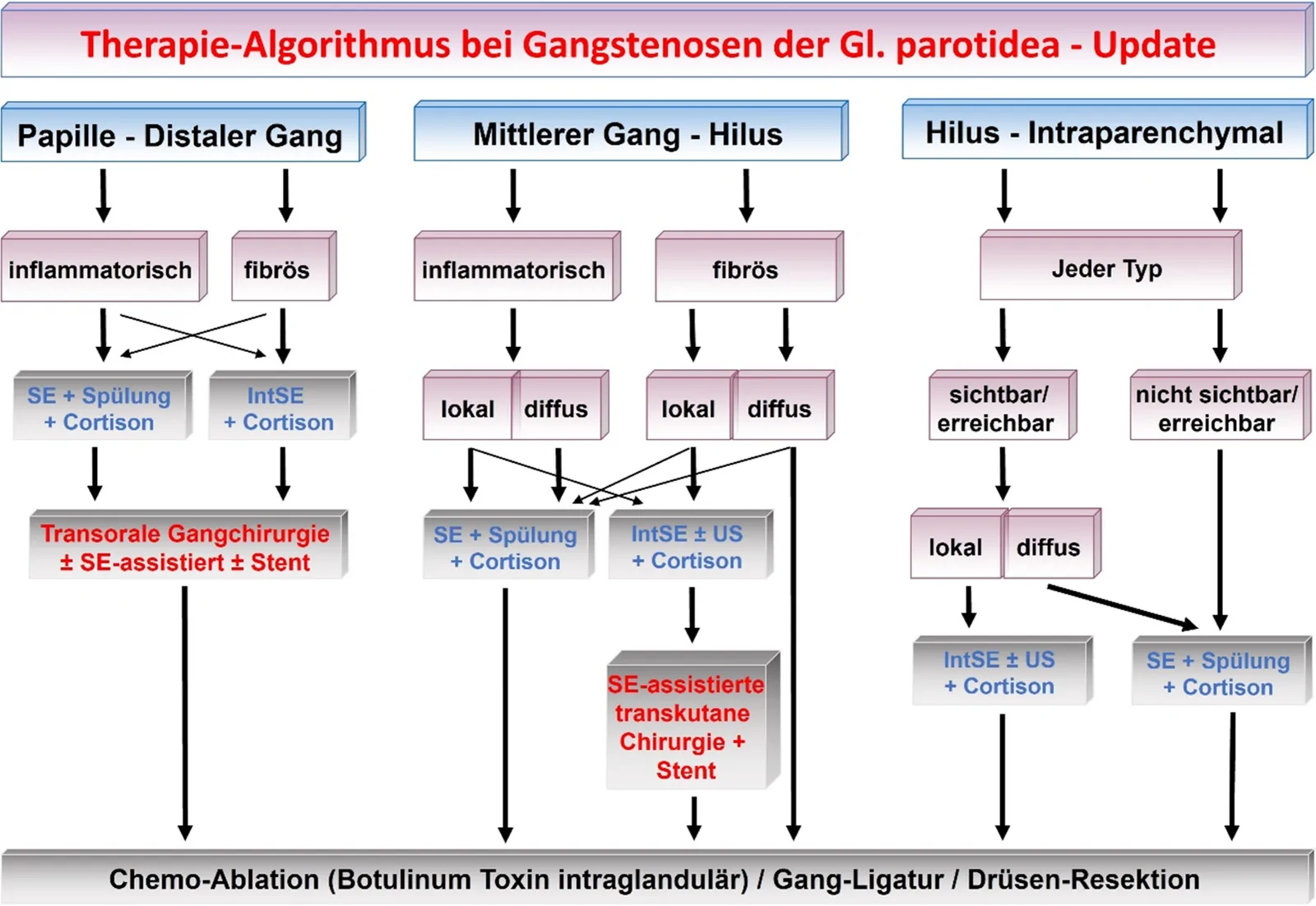
Algorithmus für die Therapie von Gangstenosen der Gl. Parotidea. *IntSE* interventionelle Sialendoskopie, *SE* Sialendoskopie, *US* Ultraschall

Implementiert man die neuesten Literaturergebnisse, ergeben sich aktualisierte Algorithmen für die Therapie der Gangstenosen der Gl. submandibularis (Abb. 7) und der Gl. parotidea (Abb. 8).

### Speichelgangverletzungen

Verletzungen des Speicheldrüsengangs sind v. a. bei der Gl. parotidea nach operativen Eingriffen, seltener nach Traumen beschrieben. Schäden des Gangsystems werden hinsichtlich ihrer Lokalisation (Zone A – intraparenchymale kleinere Gänge, Zone B – proximaler und mittlerer Hauptgang, Zone C – distaler Hauptgang bis zur Papille), das Ausmaß der Schädigung des Gangsystems (partielle Läsion der Gangwand, komplette Durchtrennung des Gangs, segmentaler Gangdefekt) oder des Parenchyms (keine, geringe, ausgeprägte Läsion des Parenchyms) eingeteilt. Für eine operative Therapie kommen die Zonen B–C, partielle bis ausgeprägte Verletzungen des Gangsystems sowie ausgeprägte Parenchymschäden infrage. Eine zusätzliche Verletzung weiterer Strukturen wie z. B. des N. facialis oder der Mandibula kann die Operation verkomplizieren. Zum Beispiel kann bei ausgeprägten Läsionen des Nervs, welche eine umfassende dynamische Rekonstruktion erfordern, eine Resektion der Drüse notwendig sein [147, 148]. Limitierte Schäden am Gangsystem der Zonen B–C können sialendoskopisch kontrolliert therapiert werden [149]. Ausgedehnte Schäden, insbesondere die komplette Durchtrennung des Ausführungsgangs oder segmentale Defekte, müssen meist offen chirurgisch therapiert werden. Die sialendoskopisch assistierte transkutane Operation stellt ein sehr geeignetes Verfahren für derartige Fälle dar. Mittels des Sialendoskops ist es möglich, den distalen Gangstumpf schnell aufzufinden und nach Auffinden des proximalen Gangstumpfs die Gewebequalität in den Gangenden genauer zu beurteilen. Nach Anfrischen der Enden erfolgt die mikrochirurgische Gangrekonstruktion i. d. R. mit Einlage eines Stents. Bisher sind einige erfolgreich therapierte Fälle publiziert worden [150–152].

### Gangentzündungen infolge inflammatorischer Erkrankungen mit oder ohne obstruktive Komponente

Die Sialendoskopie erfolgt bei Gangentzündungen in Form der therapeutischen Sialendoskopie. Sie erfolgt zur Ausspülung von Fibrinausschwitzungen und Plaques sowie zur intraduktalen Instillation von Medikamenten wie z. B. Kortison. Die wichtigsten Sialopathien, welche derzeit mittels einer therapeutischen Sialendoskopie behandelt werden, sind die idiopathische symptomatische Sialodochitis [153], die eosinophile Sialodochitis [154], das Sjögren-Syndrom [155–157], die IgG4-assoziierte Sialadenitis (eigene Daten, nicht publiziert), die durch Radiojodtherapie induzierte Sialadenitis (RAIS) [158] und die chronische rezidivierende juvenile Parotitis (cjrP) [159].

Hinsichtlich der cjrP stellt die sialendoskopisch kontrollierte Spülung einen signifikanten Fortschritt dar. In ein Review wurden 10 Studien mit insgesamt 179 Patienten eingeschlossen. Insgesamt wurde in 90 % eine Spülung mit Kortison durchgeführt und in 10 % eine interventionelle Therapie (Dilatation einer Stenose). Ein kompletter Erfolg gelang in 78 %, eine partielle Verbesserung in 22 %, die Drüse wurde in 99 % der Fälle erhalten [159]. In einer Metaanalyse, welche 7 Studien mit 120 Patienten einschloss, war die gewichtete gepoolte Erfolgsrate nach einmaliger Sialendoskopie der Patienten 73 % (95 %-KI: 64–82 %) bzw. in 81 % aller behandelten 165 Drüsen 81 % (95 %-KI: 75–87 %). Die gewichtete gepoolte Erfolgsrate nach 2 oder mehr Sialendoskopien betrug 87 % (95 %-KI: 81–93 %) [160]. In einer weiteren Metaanalyse (19 Studien, 336 Kinder) wurde über eine gewichtete gepoolte Rezidivrate von 25,8 % (95 %-KI: 21,5–30,8) nach sialendoskopisch kontrollierter Spülung berichtet [160].

Die Wertigkeit der Sialendoskopie bei RAIS nach Radiojodtherapie wurde in einem Review untersucht. Die primären Erfolgsraten lagen zwischen 50 und 100 %, die Rezidivraten zwischen 0 und 40 % [158]. In einer Metaanalyse von Beumer et al. waren die gewichteten und gepoolten Erfolgsraten nach Therapie einer cjrP (67,0 %; 95 %-KI: 53,1–78,4; 12 Studien eingeschlossen) signifikant besser als nach Therapie einer RAIS (45,8 %, 95 %-KI: 26,9–66,0; 6 Studien eingeschlossen) [85].

Auch beim Sjögren-Syndrom hat sich die sialendoskopisch kontrollierte Gangspülung mit Kochsalz bzw. Kortison als vorteilhaft erwiesen. In einem systematischen Review wurden insgesamt 8 Studien und Daten von 125 Patienten ausgewertet. Eine zumindest partielle bzw. temporäre Verbesserung der Beschwerden wurde in 95 % (95 %-KI: 90–99) beobachtet [161].

### Messung der Effekte der Sialendoskopie und sialendoskopisch assistierten Chirurgie

Insgesamt stellt die Einführung der Sialendoskopie in der Therapie von obstruktiven und inflammatorischen Erkrankungen der großen Kopfspeicheldrüsen einen bedeutenden und messbaren Fortschritt dar. Eine Metaanalyse, welche 91 Studien mit insgesamt 8218 Patienten umfasste, untersuchte die Erfolgsraten nach Sialendoskopie bzw. sialendoskopisch assistierter Chirurgie. Die gewichtete gepoolte Erfolgsrate betrug insgesamt 80,9 % (95 %-KI: 76,6–84,6) nach einer mittleren Follow-up-Dauer von 20,3 Monaten. Die Erfolgsraten waren mit 89,6 % (95 %-KI: 85,7–92,5; 48 Studien eingeschlossen) höher für die Therapie der Sialolithiasis als für die Therapie von Speichelgangstenosen mit 56,3 % (95 %-KI: 48,7–63,6; 14 Studien eingeschlossen). Die Erfolgsraten waren auch höher nach Therapie der Gl. submandibularis (88,3 %, 95 %-KI: 8,4–92,4; 25 Studien eingeschlossen) im Vergleich zur Gl. parotidea (81,2 %, 95 %-KI: 73,4–87,1; 36 Studien eingeschlossen) [85].

Der Effekt der sialendoskopisch assistierten Chirurgie wurde auch mithilfe verschiedener Fragebögen evaluiert. In einer Studie wurden 40 Patienten nach Therapie von 54 Drüsen mittels des Chronic Obstructive Sialadenitis Symptoms (COSS) Questionnaire nachuntersucht. Bei beiden Drüsen wurde eine signifikante Reduktion des durchschnittlichen Scores nach der Therapie gemessen, welche für die Gl. submandibularis (27,8; Spannweite: 38,1–10,3; *p* < 0,005) ausgeprägter war im Vergleich zur Gl. parotidea (13,6; Spannweite: 32,6–19,0; *p* < 0,0001). Patienten mit Sialolithiasis zeigten eine stärkere Verbesserung der COSS-Scores nach der Therapie als Patienten, welche wegen einer durch Radiojodtherapie induzierten oder inflammatorischen Sialadenitis therapiert worden waren. Eine Verbesserung der Symptome wurde nach Chirurgie der Sialolithiasis in 100 %, nach Therapie einer inflammatorischen Sialadenitis jedoch nur in 47 % der Fälle gemessen [162, 163].

## Fazit für die Praxis

- Bei Raumforderungen der Speicheldrüsen sind die Indikationsstellung zur Operation und die Wahl der chirurgischen Technik von der Lage und der Dignität des Tumors abhängig.
- Im Bereich der gutartigen Raumforderungen in der Gl. parotidea geht die Tendenz zu minimal invasiven und morbiditätsärmeren Eingriffen.
- Die neuesten Entwicklungen in der Chirurgie von obstruktiven und inflammatorischen Speicheldrüsenerkrankungen zeigen, dass die therapeutische und interventionelle Sialendoskopie und sialendoskopisch assistierte operative Verfahren das minimal invasive Therapiekonzept dominieren und dass nach Ausschöpfung der Therapiealgorithmen die Speicheldrüse in über 95 % aller Fälle erhalten werden kann.
